# Forecasting ICU Acute Kidney Injury with Actionable Lead Time Using Interpretable Machine Learning: Development and Multi-Center Validation

**DOI:** 10.3390/jcm15031191

**Published:** 2026-02-03

**Authors:** Abdulla Zahi Hourani, Zuzanna Jakubowska, Jolanta Małyszko

**Affiliations:** 1 Doctoral School, Medical University of Warsaw, 02-091 Warsaw, Poland; abdulla.hourani@wum.edu.pl; 2 Department of Nephrology, Dialysis and Internal Medicine, Medical University of Warsaw, 02-097 Warsaw, Poland; zuzanna.jakubowska@wum.edu.pl

**Keywords:** acute kidney injury, early warning score, intensive care unit, machine learning, XGBoost, electronic health records, external validation, calibration, SHAP

## Abstract

**Background**: Acute kidney injury (AKI) is common in intensive care and is frequently recognized late. We aimed to develop an interpretable, dynamic early warning score (EWS) to predict incident AKI within the next 24 h in ICU adult patients and to test its transportability temporally and geographically. **Methods**: We performed a retrospective cohort multi-center study using hospitalized ICU patients from MIMIC-IV and eICU-CRD databases. The outcome was incident AKI (KDIGO stage 1–3). Gradient-boosted trees (XGBoost) were trained with 10-fold cross-validation. Predictors were prespecified from the literature and finalized as 61 routinely available EHR predictors selected using SHAP ranking (spanning demographics/comorbidities, laboratory markers, vital-sign dynamics, and ICU therapies/procedures). Prespecified validations included (i) temporal validation in the COVID-19 era and (ii) geographic validation in eICU Northeast hospitals. **Results**: The development cohort included 51,833 ICU stays; temporal and geographic cohorts included 3346 and 2993 stays, respectively. Discrimination was high in internal validation (AUC 0.88, 95% CI 0.84–0.90) and remained robust temporally (AUC 0.84, 0.80–0.87) and geographically (AUC 0.82, 0.81–0.84). At a prespecified threshold, sensitivity/specificity were 0.76/0.79 (temporal) and 0.73/0.86 (geographic). Decision-curve analysis showed net benefit across clinically relevant thresholds. Key predictors reflected physiologic trajectories (e.g., mean arterial pressure dynamics), urine output, renal/metabolic markers (e.g., creatinine and BUN trends), and oxygenation dynamics (SpO_2_). **Conclusions**: A routinely updated, explainable EHR-based EWS can predict ICU AKI up to 24 h in advance with stable performance across a pandemic-era temporal shift and external geographic validation.

## 1. Background

Acute kidney injury (AKI) is a sudden decline in renal excretory function; currently defined by the Kidney Disease: Improving Global Outcomes (KDIGO-2012) criteria, it develops in up to one in five adult hospital admissions and about half of intensive care unit (ICU) encounters [1,2,3]. Far from a transient laboratory abnormality, AKI substantially increases in-hospital mortality, prolongs length of stay by several days, elevates early (30–90-day) rehospitalization risk, and accelerates the subsequent development of chronic kidney disease and cardiovascular events [2,4,5,6]. Moreover, a substantial proportion of AKI episodes go unrecognized in real time, and even when missed, are independently associated with increased short- and long-term mortality [7]. In the United States, the incremental inpatient cost attributable to AKI is estimated at USD 5.4–24 billion annually—comparable in scale to other high-burden acute conditions [4]. Despite its frequency and impact, timely recognition remains elusive: many episodes are first recognized only at stage 2 or 3, curtailing opportunities to reverse causal insults [3,8].

Older adults are particularly vulnerable: epidemiological studies show higher AKI incidence in the elderly and poorer post-AKI outcomes, consistent with age-related declines in renal repair [9]. Sex differences in AKI risk are complex. While more recent evidence suggests female sex may be renoprotective overall, with evident sexual dimorphism [10], the 2012 KDIGO guidelines note that female sex is included as a risk factor in several validated, context-specific prediction scores (e.g., contrast exposure, aminoglycosides, and cardiac surgery) [3]. Socioeconomic context further shapes who develops AKI and who recovers. A population-based cohort from England demonstrated that people in the most deprived neighborhoods had 79% higher odds of developing AKI and a 20% higher mortality after AKI than those in the least deprived areas [11]. In a contemporary U.S. hospital cohort, patients living in the most disadvantaged neighborhoods had a 10% higher adjusted risk of AKI, and rural residents had 25% higher odds of failing to recover by discharge [12]. Globally, the International Society of Nephrology (ISN) ‘0by25’ Global Snapshot reported that ~45% of AKI episodes arose in low- and lower-middle-income countries and ~8% of patients with dialysis indications did not receive it, underscoring persistent access gaps [13].

On the other hand, predictive analytics offer a potential solution, yet existing risk scores perform modestly and often arise from narrow derivation cohorts and lack proper validation [14]. Classical logistic tools such as the NHS AKI e-alert algorithm rely on serum-creatinine trajectories and therefore typically detect injury after it has begun rather than anticipating it [15]. Machine learning-based models have reported stronger discrimination; while explainable, their reliance on high-resolution physiologic signals and bespoke data structures limits transportability for the broader population and hinders routine bedside adoption [16,17,18,19]. During the COVID-19 pandemic—a pragmatic, out-of-distribution stress test—few legacy AKI scores in adults were credibly evaluated; in a prospective ICU cohort of mechanically ventilated patients with COVID-19, the adult Renal Angina Index showed only modest discrimination (area under the curve (AUC) of 0.67 at 24 h and 0.73 at 72 h) [20,21]. Reporting quality also remains a barrier: adherence to the Transparent Reporting of a Multivariable Prediction Model for Individual Prognosis or Diagnosis (TRIPOD/TRIPOD+AI) is inconsistent, external validation is uncommon, and risk-of-bias assessments with PROBAST frequently identify concerns [14,22,23].

Concurrently, hospital electronic health records (EHRs) have matured into longitudinal, structured repositories that capture hundreds of variables from different modalities, enabling models to extract prognostic signals while preserving explainability. When thoughtfully integrated with gradient-boosted trees, such systems can combine non-linear pattern recognition with the calibration strength and computational efficiency of classical models. These methodological developments are timely because preventing AKI requires predictions that are both early and actionable. A 24 h horizon at the start of admission aligns with clinically meaningful decision points: whether to pursue contrast-enhanced imaging, optimize fluid resuscitation and hemodynamic targets, or review potentially nephrotoxic antibiotics and renin–angiotensin inhibitors [3,8,24]. Equally important is an implementation pathway that embeds seamlessly into existing EHR workflows, furnishes clinicians with interpretable explanations, and avoids the data-privacy barriers that accompany cloud-hosted services [25].

Accordingly, we developed a prognostic, dynamic early-warning score built for clinicians to predict incident AKI within the next 24 h for ICU patients and which updates their risk throughout their stay. To rigorously test generalizability, we performed two prespecified validations: (i) a temporal validation in a held-out MIMIC-IV cohort admitted during 2020–2022, capturing the COVID-19 pandemic as a real-world stress test, and (ii) a geographic external validation in eICU hospitals from the U.S. Northeast region (seven hospitals). The model remained well-calibrated and suitable for bedside use without reliance on high-frequency waveforms or bespoke data feeds.

## 2. Materials and Methods

### 2.1. Data Sources and Cohort Assembly

This retrospective cohort study used the Medical Information Mart for Intensive Care (MIMIC-IV) and the Electronic Intensive Care Unit Collaborative Research Database (eICU-CRD) datasets [26,27,28,29,30]. These datasets integrate hospital-wide electronic health records with an intensive care unit (ICU) clinical information system. They provide detailed demographics, vital signs, laboratory measurements, medication exposures, and procedural information. MIMIC-IV encompasses 94,458 ICU stays from Beth Israel Deaconess Medical Center between 2008 and 2022, while eICU-CRD captures 200,859 ICU stays from 208 hospitals across the United States during 2014–2015. 

We constructed a rolling-window cohort from all ICU stays satisfying prespecified inclusion criteria. We censored each stay at the first incident AKI to avoid multiple outcomes per stay. Adults aged 18–88 years were eligible; entries coded as “>89” were excluded because their ages were masked for privacy. We removed stays where kidney replacement therapy was already in use at ICU entry or where acute kidney injury (AKI) had already developed by the first window. Stays that were missing end-stage kidney disease or baseline AKI status were excluded. Figure S1 in the Supplementary Methods outlines the exclusion criteria in detail. We then defined two held-out test frames to assess temporal and geographic generalization: (i) a MIMIC-IV cohort comprising ICU stays with all admission dates from 2020 to 2022, overlapping the COVID-19 pandemic, and (ii) an eICU cohort restricted to hospitals in the U.S. Northeast region (a total of 7). All remaining eligible stays from MIMIC-IV and eICU constituted the training dataset (MIMIC-IV 2008–2019, and eICU hospitals in the Midwest, West, and South regions. Internal validation was performed using 10-fold cross-validation. All prediction windows from a patient (across different stays) were assigned to the same cross-validation fold and data partition to prevent information leakage.

### 2.2. Rolling-Window Prediction Framework and Outcome Definition

**Figure 1:**
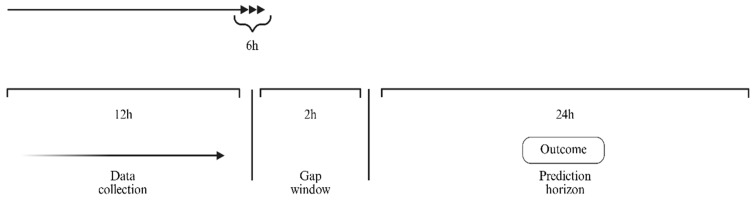
Windowing scheme for model predictions. Each ICU stay was expanded into overlapping windows consisting of a 12 h feature window, a 2 h gap to prevent information leakage, and a 24 h outcome horizon; predictions refreshed every 6 h. The outcome is incident KDIGO stage 1–3 AKI within the prediction horizon.

Each ICU stay was expanded into overlapping windows: a 12 h feature window, a 2 h gap to prevent leakage, and a 24 h outcome horizon; windows (predictions) updated every 6 h (Figure 1). We compared several window/gap/horizon configurations and found this pattern best aligned with variable availability and routine measurement frequency in the training data. To balance situational awareness with alert burden, the 6 h stride yields at most two risk refreshes per patient during a standard 12 h shift, aligning with typical rounding and handoff cycles. By targeting a 24 h horizon, the score surfaces risk early enough for preventive and renal-protective actions, aligning with KDIGO’s emphasis on short-term AKI dynamics.

The primary endpoint was binary incident AKI (KDIGO stages 1–3) occurring after the end of each prediction cutoff (i.e., after the 12 h feature window and the subsequent 2 h gap) and within the following 24 h outcome horizon. AKI was operationalized using KDIGO criteria based on serum creatinine (SCr), urine output (UO), and renal replacement therapy (RRT), implemented using the MIMIC-IV-derived KDIGO SQL concept code (MIT-LCP mimic-code repository) with an explicitly time-respecting label construction. For the creatinine criteria, at each laboratory timestamp t, we computed a time-varying baseline SCr using only measurements available prior to *t*: (i) the minimum SCr in the preceding 48 h (to evaluate the ≥0.3 mg/dL rise criterion), and (ii) the minimum SCr in the preceding 7 days (to evaluate the 1.5×/2×/3× relative rise criteria). When no prior SCr existed within the relevant lookback window, the creatinine criterion could not be computed. UO criteria were derived by aggregating charted urine volumes into hourly totals, computing rolling UO rates over the prior 6, 12, and 24 h, and normalizing to body weight (kg) recorded within each rate. If weight was unavailable, UO-based staging was treated as missing. At any time t, the KDIGO stage was the maximum of the creatinine-based stage, urine-output-based stage, and stage 3 if RRT had been initiated. The AKI onset timestamp was defined as the earliest time any KDIGO criterion was first met. To distinguish incident from prevalent AKI at ICU entry, we excluded stays that met KDIGO criteria before the first eligible prediction cutoff and censored subsequent windows after the first onset.

### 2.3. Feature Selection and Processing

All preprocessing and feature engineering were performed exclusively on the training data, and the learned parameters were applied to the corresponding validation/test sets to prevent data leakage. Candidate predictors came from the nephrology and critical-care literature and included demographics, comorbidities, vital signs, laboratory tests, medication exposures, ventilatory support, and procedural indicators. Continuous time series were summarized using various representations such as averages, standard deviations, and slopes. Categorical variables were one-hot encoded. Missing values were not imputed, as tree-based models can natively handle missingness. Leaving values missing preserves the true sparsity structure of the EHR time series, which is predictive in its own right. Treating absence as a feature lets the model exploit ordering patterns (e.g., labs obtained only when concern is high) that imputation would dilute. Predictors with more than 50% missingness were discarded, and near-zero-variance predictors were removed. Pearson and Spearman tests were used to assess collinearity. Descriptive analyses were performed at the prediction-window level, stratified by whether AKI occurred within the subsequent 24 h horizon—categorical variables were represented in counts and percentages; on the other hand, continuous variables were represented in medians and interquartile ranges. Between-cohort differences were quantified using absolute standardized mean differences (ASMD), weighted across windows.

### 2.4. Model Development and Feature Selection

We trained gradient-boosted decision trees using the XGBoost framework [31]. This approach was chosen because it consistently outperforms linear and deep baselines in heterogeneous EHR tabular data, while natively handling missingness and non-linear interactions. A grid search for hyperparameter tuning was performed using 10-fold cross-validation at the patient level, which preserved clustering of windows of stays and patients, and allowed leakage-free evaluation during tuning. Monotonic constraints (Table S3) were applied to clinically directional predictors to encode established physiology (e.g., higher creatinine/BUN and vasopressor dose should not decrease AKI risk, whereas higher urine output should not increase risk), thereby improving biological plausibility, reducing spurious sign-flips driven by noise or missingness, and stabilizing generalization across patients and time windows. To reduce the number of features needed for inference, we calculated SHAP values of features during cross-validation of the final model, and selected 61 features with which we rebuilt the same model, noting an insignificant marginal drop in performance.

### 2.5. Performance Evaluation

Discrimination was assessed on cross-validation, temporal, and geographical sets by AUC and area under the precision-recall curve (AUPRC), supplemented by F1- and F2-scores, recall, specificity, precision, balanced accuracy, and the Matthews correlation coefficient. Point estimates and 95% confidence intervals were computed using a 2000-sample bootstrap with clustering at the patient level. Calibration was assessed through the calibration intercept and slope, Brier score, and reliability plots. We also assessed calibration using calibration in the large (CITL) and expected calibration error (ECE). We additionally applied Platt scaling to the booster’s predictions using out-of-fold predictions. In addition, we generated a calibration curve to visualize agreement between predicted and observed risks and conducted decision curve analysis (DCA) to evaluate net clinical benefit across clinically relevant threshold probabilities. Predictor-level importance was further explored using SHAP values [32].

Furthermore, we also reported event-level detection performance based on the first alert preceding an AKI event, including detection rates at prespecified lead-time thresholds. Detection-rate bins were summarized across predefined lead-time windows (>6, >12, >18, >24) and lead-time bins, and false-positive alerts were categorized by time to a subsequent AKI event (24–48 h, 48–72 h, >72 h, or no subsequent AKI). We additionally quantified alert burden using total alerts, alerts per patient-day and per 12 h shift, time under alert (raw and length-of-stay capped), repeat-alert rate, and the proportion of stays with any or ≥2 alerts.

### 2.6. Sensitivity Analyses and Subgroup Assessments

We evaluate the model’s performance against different scenarios. First, we assess the difference in performance between the presence and absence of monotonic constraints. Second, we compare the model’s performance using the selected feature set vs. all. Third, we evaluate the model’s performance using different AKI criteria, i.e., creatinine-based or urine-output-based. We then evaluate the model’s performance against different percentages of data missingness.

We assessed heterogeneity of discrimination in predefined subgroups within the temporal and external validation cohorts, stratifying by age (<50, 50–74, and ≥75), sex, race (harmonized categories: Asian, Black, White, and Hispanic), and comorbidities (chronic kidney disease, acute myocardial infarction, heart failure, diabetes, cirrhosis, hypertension, sepsis, contrast, and COVID-19). AUCs were estimated with confidence intervals using a cluster-based 1000 bootstrap; differences between subgroups were compared using DeLong tests with Bonferroni correction applied across the subgroup comparisons. We further reported false and true negative and positive rates and calibration metrics for age, sex, and race subgroups across both validation sets.

### 2.7. Data Statement and Ethics

This study used fully de-identified patient data. Both the eICU-CRD and MIMIC-IV are publicly available critical care datasets that have undergone institutional review board (IRB) review and approval for data collection and sharing. Access to these resources is provided only after completion of the required training and execution of a data use agreement. All records are distributed under de-identification procedures consistent with the Health Insurance Portability and Accountability Act (HIPAA) Safe Harbor standard and therefore contain no direct identifiers. Because the analyses were conducted exclusively on de-identified data, the work does not constitute human-subject research involving identifiable private information, and informed consent was not required. The study was performed in accordance with applicable ethical and regulatory standards for the use of de-identified health data.

Detailed data processing, model development, training procedures, hyperparameter optimization, and sensitivity analyses are described in depth in the Supplementary Methods.

## 3. Results

### 3.1. Cohort Characteristics and Descriptive Analyses

Table 1 summarizes cohort characteristics at the prediction-window level (i.e., all eligible 6-hourly rolling windows used for model development/validation). These windows were generated from 51,833 unique ICU stays in the development cohort and 3346 and 2993 unique ICU stays in the temporal and external validation cohorts, respectively. Incident AKI (KDIGO stage 1–3 within 24 h) occurred in 11.9% of development windows, 17.1% of temporal validation windows, and 14.2% of external validation windows, indicating a higher event rate in both validation settings. Sex distribution was similar across cohorts (45.9% vs. 47.9% vs. 45.0% female; temporal ASMD = 0.06; external ASMD = 0.03). Median age was slightly lower in the external validation set (66 vs. 65 vs. 63 years; external ASMD = 0.14). ICU length of stay was longer in the temporal cohort (3.6 vs. 4.1 vs. 3.8 days; temporal ASMD = 0.19). Substantial compositional shifts were observed in race/ethnicity: compared with development, the temporal cohort had fewer White patients, and more patients labeled “Other race” (ASMD up to 0.39), while the external cohort had markedly more White and fewer Black/Hispanic patients (ASMD up to 0.61). Unit type also differed: the temporal cohort was enriched for Neurology ICU admissions (49.3% vs. 13.8%; ASMD = 0.68), whereas the external site showed fewer cardiothoracic and medical ICU admissions and mixed ICU care, and more surgical/trauma admissions. Many comorbidities were broadly comparable (ASMD ≤ 0.10), but several conditions were more prevalent in the temporal cohort, e.g., shock (24.7% vs. 20.3%; ASMD = 0.33), while others were less prevalent, such as sepsis (13.8% vs. 17.4% in development; ASMD = 0.38, reflecting lower rates in temporal than development), as well as in the external cohort (shock 36.5%, ASMD = 0.31; sepsis 19.2%, ASMD = 0.43). Hypertension was more common in the temporal cohort (60.2%; ASMD = 0.06) but less common externally (46.7%; ASMD = 0.24). Atrial fibrillation was slightly higher in the temporal cohort (18.5%; ASMD = 0.04), while chronic kidney disease was lower (5.0%; ASMD = 0.20) relative to the development cohort.

Several laboratory distributions shifted meaningfully. The anion gap was notably lower in both validation cohorts relative to development (median 11.0 temporally and 9.5 externally vs. 13.0), with large imbalances (temporal ASMD = 0.65; external ASMD = 0.78). The temporal cohort had lower MCHC (ASMD = 0.38) and slightly higher MCV (ASMD = 0.16). External validation showed modest differences in creatinine and BUN (ASMD ≈ 0.12–0.14) and a small shift in hemoglobin (ASMD = 0.06). Overall, most other labs had small-to-moderate imbalances. Temporal validation exhibited higher mean arterial pressure (82.3 vs. 78.6 mmHg; ASMD = 0.30) and lower heart rate (ASMD = 0.18) compared with development, alongside modestly lower SpO_2_ (ASMD = 0.18). Urine output in the 12 h prior to prediction was lower temporally (median 850 mL; ASMD = 0.24) but similar externally (median 1025 mL; ASMD = 0.03). Temperature was slightly higher externally (ASMD = 0.21). Treatment patterns varied across settings. The external cohort had higher use of ACEi/ARB (9.1% vs. 3.3%; ASMD = 0.27), statins (11.4% vs. 5.0%; ASMD = 0.03), and insulin (28.3% vs. 14.8%; ASMD = 0.10), and lower rates of CABG (0.7% vs. 1.6%; ASMD = 0.31) and valve surgery (0.7% vs. 1.1%; ASMD = 0.26), consistent with different case-mix and practice patterns. Phenylephrine exposure was more frequent in both validation cohorts (≈7.0% vs. 3.7%), with a large imbalance externally (ASMD = 0.39). Norepinephrine was slightly less common temporally (ASMD = 0.19). Contrast administration was far less frequent outside the development cohort (ASMD ≈ 0.20 externally).

As expected, comorbidities and most treatments had negligible missingness. Laboratory and vital sign variables showed varying missingness across sites and time, typically ~25–44% for several hematology and chemistry labs and ≤7% for core vital signs. Height and weight had low-to-moderate missingness (≈0–12%) in development and external validation cohorts, with higher rates in the temporal cohort for height. Percent missing for each variable and cohort is reported in Table 1 and was accounted for in model training and evaluation. Overall, most variables showed small imbalances between development and validation cohorts (ASMD ≤ 0.10). However, meaningful shifts were observed in a limited set of features; most notably race/ethnicity, ICU service mix (especially Neurology ICU in temporal validation), anion gap, mean arterial pressure, urine output, markers of infection/shock, and select treatments/procedures (e.g., ACEi/ARB, phenylephrine, CABG). These shifts likely reflect differences in case mix, measurement practices, and therapeutic patterns across time and geography. The observed heterogeneity underscores the importance of reporting transportability results alongside internal validation.

### 3.2. Discriminative Performance

**Figure 2:**
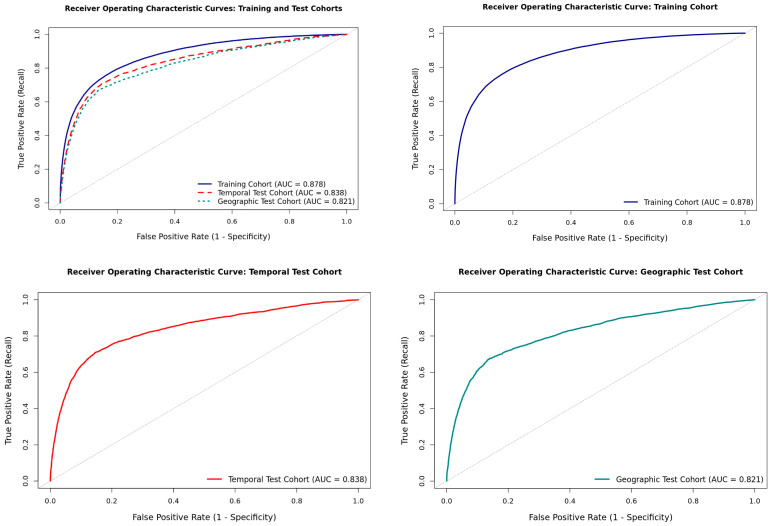
Discrimination of the final model (AUC) for 24 h AKI prediction in training (10-fold cross-validation), temporal validation (MIMIC-IV 2020–2022), and external validation (eICU Northeast). Abbreviations: AUC: area under the curve.

Our model demonstrated high discrimination for predicting acute kidney injury (AKI) up to 24 h before onset (Table 2 and Figure 2). In internal cross-validation, the model achieved an AUC of 0.88 (95% CI, 0.84–0.90). Discrimination remained robust in the temporal validation cohort (2020–2022 admissions), with AUC 0.84 (0.80–0.87)—a modest absolute decrease of 0.04 relative to development—and in the external geographic cohort with AUC 0.82 (0.81–0.84) (Δ = 0.06 vs. development). The corresponding AUPRC was 0.60 in the temporal cohort and 0.53 (0.50–0.57) in the geographic cohort, consistent with performance under class imbalance. To prioritize sensitivity, we prespecified a decision threshold of 0.125, selected in development to maximize the F2-score and corroborated by decision-curve analysis. At this high-sensitivity operating point, the temporal cohort yielded a sensitivity (recall) of 0.76 (0.73–0.80) and specificity of 0.79 (0.76–0.83), effectively identifying approximately three in four impending AKI events 24 h in advance. For external geographic validation, performance at the same threshold was a recall of 0.73 (0.67–0.78) with a specificity of 0.86 (0.83–0.89). Balanced accuracy was 0.82 (0.78–0.86) in cross-validation and remained high in temporal and geographic cohorts—0.78 (0.76–0.83) and 0.80 (0.78–0.84), respectively. F1- and F2-scores were comparable between development and validation, underscoring the stability of the selected operating point across settings. 

Additional lead-time analyses (Figure S6) showed that 30.6% of temporal-validation events and 32.8% of external-validation events were detected with >6 h lead time, declining to 24.4%/27.3% at >12 h, 20.1%/23.2% at >18 h, and 16.0%/19.4% at >24 h. Alert burden metrics (Table S7) recorded 4252 temporal and 3538 external alerts, corresponding to 0.19 and 0.21 alerts per patient/day (0.096 and 0.103 per 12 h shift). Repeat alerts accounted for 16.1% and 12.4% of alerts, and 20.95% and 15.77% of stays had ≥2 alerts. False-positive analytical results are detailed in the Supplementary Methods (Table S8).

### 3.3. Calibration, Decision Curve Analysis, and Key Predictors

Across datasets, overall accuracy remained stable, with MCC 0.50 (0.46–0.55) in development, 0.47 (0.43–0.49) in temporal validation, and 0.50 (0.44–0.54) in external validation; corresponding Brier scores were 0.07 (0.04–0.10), 0.10 (0.07–0.13), and 0.09 (0.07–0.14), respectively. Calibration was close to ideal in temporal validation before re-calibration (slope 0.94, CITL 0.074, and ECE 0.008) and improved further after Platt scaling (slope 1.03, CITL 0.008, and ECE 0.005), with calibration curves closely tracking the 45° reference (Figure S2). External calibration initially showed an intercept shift with mild slope miscalibration (slope 1.038, CITL 0.361, and ECE 0.030); recalibration brought the slope to 1.004, CITL to 0.057, and ECE to 0.008, improving agreement between predicted and observed risks (Figure S2). 

Decision curve analysis (Figure S3) demonstrated that using the EWS to trigger a kidney-protective response yielded higher net benefit than either intervening in all windows (“treat all”) or none (“treat none”) across a clinically relevant range of threshold probabilities in both the temporal and external validation cohorts. Importantly, net benefit should be interpreted as true positives minus false positives weighted by the threshold-derived harm/benefit trade-off (weight = pt/(1 − pt)); it does not generally translate directly into “additional true-positive alerts per 100 evaluations” without that weighting. Because our intended operating point was prespecified at 0.125 (12.5%)—chosen in development to maximize the F2-score (sensitivity-prioritized) and corroborated by DCA—we focus interpretation on this threshold. At pt = 0.125, the implied false-positive weight is 0.125/0.875 = 0.143, corresponding to a policy in which the harm of an unnecessary response is considered substantially lower than the harm of missing an impending AKI. Using the observed window-level AKI incidence (17.1% temporal; 14.2% external) and the operating-point confusion-matrix metrics at pt = 0.125, the implied net benefit of the EWS at 12.5% was 0.109 in temporal validation and 0.088 in external validation, exceeding both “treat none” (0 by definition) and “treat all” (0.053 temporal; 0.019 external) at the same threshold. Expressed as a net reduction in unnecessary interventions relative to “treat all”, this corresponds to avoiding approximately 39.5 (temporal) and 47.7 (external) unnecessary responses per 100 prediction windows at the same threshold-defined trade-off. At this operating point, discrimination–classification performance remained stable across cohorts (temporal: recall 0.76 (0.73–0.80), specificity 0.79 (0.76–0.83), precision 0.47 (0.43–0.54), and F2 0.68 (0.62–0.73); external: recall 0.73 (0.67–0.78), specificity 0.86 (0.83–0.89), precision 0.48 (0.42–0.54), and F2 0.66 (0.61–0.68)). The choice of 12.5% is consistent with an intended response policy centered on low-harm, kidney-protective actions (e.g., medication/nephrotoxin review and dose adjustment, reassessment of hemodynamics/volume status and perfusion targets, and closer monitoring), where the principal “cost” of false positives is additional clinical workload and potential alert fatigue rather than invasive therapy. 

**Figure 3:**
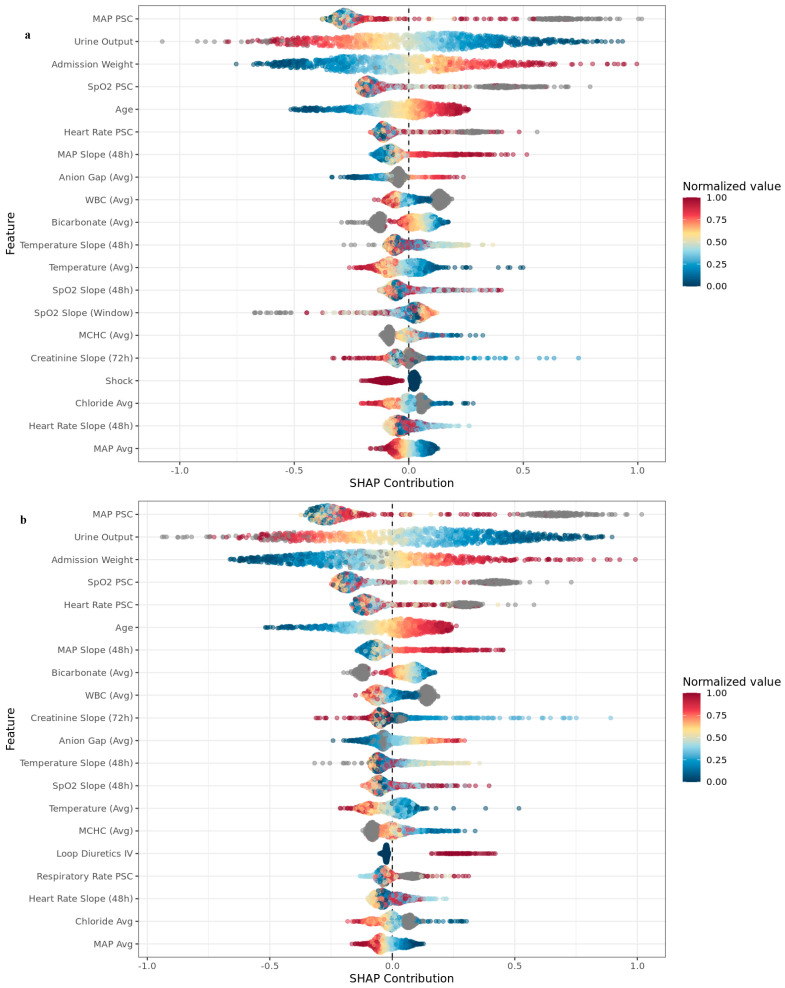
SHAP summary of the top 20 predictors for (**a**) external validation (Northeast) and (**b**) temporal validation (COVID-19 era). Each dot is a prediction window; the x-axis shows the SHAP contribution to the model output for 24 h AKI risk (positive increases predicted risk). Features are ordered by mean absolute SHAP. Dot color indicates normalized feature value (min–max scaled within each cohort; blue = low, red = high). PSC = piecewise slope change.

Model interpretability using SHAP values (Figure 3) and variable-importance analyses (Figure S4) indicated that physiologic trajectories were the principal drivers of predicted risk: mean arterial pressure piecewise slope change was the largest contributor (downward trends increased risk; stable/recovering patterns were protective), and lower urine output showed strong monotonic risk-increasing effects. Older age and higher admission weight tended to increase risk. Worsening oxygenation dynamics (SpO_2_ piecewise and window slopes) and adverse heart-rate dynamics (piecewise change and 48 h slope) elevated risk, emphasizing instability over isolated measurements. Renal and metabolic markers were directionally coherent: rising creatinine (72 h slope) and higher BUN increased risk; lower bicarbonate and higher anion gap were deleterious; and higher WBC added to the inflammatory risk. Temperature level and 48 h slope showed bidirectional effects, rising glucose over 72 h modestly increased risk, and hematologic indices and ICH/SAH contributed the least.

### 3.4. Subgroup Performance and Sensitivity Analyses

Across two independent validation sets (temporal and external), using DeLong’s test, the EWS showed stable discrimination across most subgroups (Table 3). AUCs by age and sex were similar (temporal: 50–74, 0.83 vs. 0.81, *p* = 0.084; ≥75, 0.84 vs. 0.81, *p* = 0.061; female, 0.84 vs. 0.83, *p* = 0.372; external: 50–74, 0.82 vs. 0.81, *p* = 0.800; ≥75, 0.82 vs. 0.81, *p* = 0.726; female, 0.82 vs. 0.82, *p* = 0.56), and performance was comparable across racial/ethnic groups (e.g., Black: temporal 0.81 vs. 0.84, *p* = 0.142; external 0.82 vs. 0.82, *p* = 0.902). The EWS remained robust for CKD (temporal 0.86 vs. 0.84, *p* = 0.145; external 0.80 vs. 0.82, *p* = 0.181), diabetes (temporal 0.84 vs. 0.84, *p* = 0.973; external 0.81 vs. 0.82, *p* = 0.614), hypertension (temporal 0.84 vs. 0.83, *p* = 0.256; external 0.83 vs. 0.81, *p* = 0.324), and contrast exposure (temporal 0.84 vs. 0.84, *p* = 0.433; external 0.85 vs. 0.82, *p* = 0.646). Three exceptions reached statistical significance: lower AUC in sepsis in both cohorts (temporal 0.79 vs. 0.83, *p* = 0.004; external 0.79 vs. 0.82, *p* = 0.030), higher AUC in temporal acute MI (0.88 vs. 0.85, *p* = 0.024), and lower AUC in external cirrhosis (0.79 vs. 0.82, *p* = 0.046); differences for AHF were borderline only (external *p* = 0.066). In the temporal cohort, COVID-19 results were similar to the reference (0.82 vs. 0.84, *p* = 0.143). At the prespecified alert threshold (predicted AKI risk ≥0.125), threshold-dependent performance was consistent across key demographic strata (Table S9). Sensitivity was similar by sex (female 75.5% vs. male 74.2%), with comparable false-negative rates (24.5% vs. 25.8%) and specificity (83.4% vs. 82.8%). Across age groups, sensitivity remained stable (76.2% in <50, 75.4% in 50–69, 74.8% in ≥70), while specificity was comparable (83.8%, 83.2%, and 82.9%, respectively), with modestly higher miscalibration in older patients (CITL +0.08; slope 0.92). Threshold performance was also broadly similar across race/ethnicity, with sensitivity ranging from 73.2% to 76.0% and specificity from 83.0% to 85.4%; calibration was acceptable overall (slopes 0.90–0.98), with the largest negative intercept shift in Hispanic patients (CITL −0.15). Across all subgroups, Brier scores were low (0.074–0.108), supporting stable overall accuracy at the chosen operating point. Sensitivity analyses results are presented in the Supplementary Methods.

## 4. Discussion

### 4.1. Principal Findings in Context

In this multi-center study, we developed a transparent early warning score (EWS) for predicting AKI up to 24 h in advance, and we demonstrated its robustness across different hospitals, time periods, and patient subgroups. The model achieved high discrimination (AUC 0.88 in development) and remained strong under two rigorous validation tests: a temporal validation during the COVID-19 pandemic (AUC 0.84) and a geographic external validation of seven hospitals in the Northeastern region of the US (AUC 0.82). The modest declines of 0.04–0.06 in AUC indicate excellent transportability despite substantial shifts in case-mix and practice patterns. Notably, performance stayed strong even in the pandemic cohort, effectively serving as a real-world out-of-distribution stress test. This resilience contrasts with many legacy AKI risk tools that struggled with COVID-19. For example, the Renal Angina Index—a prior score for early AKI risk—achieved only modest accuracy in COVID-19 ICU patients (AUC ~0.67 at 24 h), underscoring that our EWS maintained far better predictive power under similarly challenging conditions [21]. More broadly, early COVID studies highlighted that many rapidly developed prognostic models were inadequately validated: a living review found 145 COVID-19 prediction models (for mortality and progression) all at high risk of bias, and none could be recommended for clinical use [33]. These examples underscore how conventional scores and rushed models underperformed under pandemic pressures, and the generalizability of our predictions suggests that our model is capturing fundamental patient-risk signals rather than overfitting to a narrow population. Geographic external validation across seven hospitals in the U.S. Northeast confirmed possible durable performance (AUC 0.82) despite marked shifts in service mix, laboratory distributions, and treatment patterns. Calibration remained near-ideal after minor recalibration, and decision-curve analysis demonstrated consistent net clinical benefit across clinically relevant thresholds. Collectively, these results indicate a potentially genuine transportability across geography and practice environments, supporting readiness for pragmatic deployment. By providing stable risk estimates across such varied settings, the EWS can underpin everyday choices about how closely to monitor the kidneys, when to escalate supportive care, and which patients should be prioritized for early kidney-protective strategies.

At the sensitivity-optimized threshold, the model in development and validation cohorts detected roughly three-quarters of impending AKI cases 24 h in advance, with about half of alerts representing true positives. These results are clinically significant given the high incidence and burden of AKI—which occurs in up to 50% of critical-care patients [2] and is associated with increased mortality, prolonged hospitalization, long-term renal impairment, and substantial costs [4,6]. By detecting AKI early (often before it reaches severe KDIGO stage 2–3), our model could enable timely interventions to mitigate kidney injury. In practical terms, this advance warning window can be used to reassess volume status, re-evaluate nephrotoxic medications, and adjust hemodynamic targets at a moment when kidney injury is still potentially reversible. These early bedside actions mirror contemporary AKI treatment principles, where supportive care focuses on individualized hemodynamic optimization, careful fluid strategy, nephrotoxin stewardship, and medication dose adjustment while the underlying cause is addressed [34]. In addition, coupling an alert to a structured escalation pathway can standardize early nephrology input and prompt assessment for RRT indications (e.g., refractory acidosis, dangerous electrolyte derangements, diuretic-resistant volume overload, or uremic complications), rather than initiating RRT “early” in the absence of clear indications [34]. This performance is on par with more complex deep-learning systems such as the Google DeepMind continuous AKI predictor, which achieved an AUC ~0.92 with a 48 h horizon. However, whereas the DeepMind model captured 55.8% of in-hospital AKI events at a 2:1 false-alert ratio [25], our model—using only routinely available ICU inputs and a shorter 24 h horizon—was able to detect a greater fraction of cases (up to ~79%) with fewer false alarms per true alert. Because it relies solely on standard ICU data streams, the score can be embedded into existing rounds and checklists without requiring new hardware or complex data feeds, lowering the barrier to using it to evaluate AKI risk routinely at the bedside. In summary, our EHR-based AKI early-warning score attains state-of-the-art discrimination and offers clinically actionable lead time, underscoring its potential value in improving the recognition and management of AKI in critical care.

Reassuringly, the EWS maintained discrimination across diverse subgroups in our subgroup analyses. We observed no significant performance drop between female vs. male patients or across major racial categories; however, estimates for the Hispanic subgroup in the temporal validation cohort were based on relatively fewer observations and should therefore be interpreted cautiously. This is encouraging given known sex and race disparities in AKI epidemiology and care, and it suggests the model does not inherit overt bias from these variables [9,10,11,12,13]. These subgroup findings are clinically important because they support the use of a single, unified AKI warning strategy across diverse ICU populations without systematically disadvantaging women, older adults, or racial minorities. In fact, performance was similar by sex (AUC ~0.82–0.84 in both women and men), suggesting the model does not systematically overestimate risk in women despite sex-related differences in AKI epidemiology that vary by clinical context. We also saw stable performance in older adults, a critical target population for AKI prevention. Only a few extreme subgroups showed slightly lower AUC (e.g., patients with sepsis or liver cirrhosis had AUC ~0.79 on external validation), likely reflecting the inherent difficulty of prediction in these high-risk scenarios where AKI incidence is very high. Overall, the consistency of performance across subpopulations and the turbulent COVID-19 period attests to the robustness of our approach. It reinforces that our model’s features and training strategy captured broad physiological signals that remained valid despite shifts in case mix. This stability means that the same alert threshold and response pathway can be applied across most ICU beds, simplifying protocols and reducing the risk that particular demographic groups receive weaker kidney-protective care. In practice, quality-improvement programs and AKI prevention pathways built around the EWS could be implemented at the unit or hospital level, unlike previous models focusing on small subgroups, while local monitoring can ensure that no new inequities emerge over time.

### 4.2. Comparison with Prior AKI Prediction Tools

Our findings compare favorably with previous AKI prediction efforts in both performance and scope. Traditional rules and e-alerts—exemplified by the NHS creatinine-trajectory algorithm—are inherently reactive, typically signaling injury only after it has begun, with limited opportunity to avert progression [15]. Earlier machine-learning efforts using first-day ICU data improved discrimination but remained single-shot and constrained by narrower feature sets; for example, a MIMIC-III model reported mean AUC ≈ 0.78 for 72 h AKI without continuous updating [16]. More generally, many static ML scores were derived at single centers or from bespoke high-frequency inputs, which hampers transportability and routine bedside use. Continuous, real-time scores have since advanced the field. A deep-learning approach achieved state-of-the-art 48 h prediction in a veteran population but showed performance attenuation and sex-related miscalibration upon external application, necessitating retraining on more diverse data [25,35]. In parallel, simple, interpretable, real-time EHR models have demonstrated strong performance in general hospital cohorts using parsimonious routine variables and multi-site validation [36], while perioperative dynamic tools that leverage minute-level intraoperative signals (e.g., hypotension depth/duration) have improved post-operative AKI prediction in surgical settings, albeit with dependence on high-resolution monitoring infrastructure [17,18]. Within this continuum, our ICU-focused EWS updates risk every 6 h using rolling windows, trajectory features (e.g., piecewise MAP/SpO_2_ change), and regularized boosting with monotonicity constraints, yielding materially higher discrimination than earlier ICU scores and maintaining AUC ≈ 0.82–0.84 on temporal and external validations from routine EHR data. By pairing dynamic prediction with careful calibration and predefined operating thresholds, the score balances sensitivity and precision, remains implementable across institutions, and avoids the portability pitfalls that have limited both static ML models and some high-frequency continuous approaches. Taken together, these comparisons suggest that our EWS occupies a pragmatic middle ground: sophisticated enough to capture dynamic physiology, yet simple and robust enough to be deployed as a standard component of ICU surveillance rather than a bespoke research system. However, cross-study performance comparisons (including to the Google DeepMind continuous AKI predictor) should be interpreted as qualitative context rather than equivalence, because prediction horizons, input modalities/dimensionality, risk update frequency, AKI definitions, and evaluation/alerting protocols differ across studies. We reference these prior systems because they represent the strongest and closest available benchmarks for continuously updated AKI risk estimation from EHR data, but definitive claims require head-to-head evaluation on the same cohort with matched horizons, thresholds, and alerting policies.

COVID-19 brought a high and heterogeneous burden of AKI across hospitals and ICUs, with AKI strongly linked to adverse outcomes; proposed mechanisms span hemodynamic instability, inflammatory injury, microvascular thrombosis, and nephrotoxin exposure. This volatility stressed legacy risk tools and underscored the need for adaptive, context-aware prediction during surges [20]. Compared to prior ICU-based AKI scores, our EWS provides both earlier and more individualized predictions. The Renal Angina Index and other point-based scores offer a single snapshot risk estimate and perform variably in adults (and, as noted, poorly in COVID-19 cohorts) [21]. In contrast, our model updates risk every 6 h, allowing clinicians to see trends and respond to worsening trajectories. This dynamic approach aligns with the evolving nature of critical illness. Additionally, by leveraging routine EHR data (vitals, labs, comorbidities, and treatments) rather than proprietary high-frequency data streams, our EWS is inherently more transportable. It can be implemented with data available in most hospital information systems, which is a key advantage for scalability. This design choice, focusing on widely captured variables was deliberate to avoid the “data richness” trap that hinders many promising ICU ML models from wider use [37]. In summary, our EWS not only delivers high discrimination on par with the best reported models, but it does so with greater transparency and ease of integration, addressing several limitations noted in the AKI prediction literature.

### 4.3. Model Explainability and Clinical Utility

A defining feature of our approach is the emphasis on feature attribution and interpretability. We employed explainable gradient boosting and SHAP values to ensure that the model’s predictions can be understood in clinical terms [38]. The leading drivers of risk identified by our model—such as declining mean arterial pressure, drops in urine output, rising creatinine/BUN, and worsening oxygenation—are all intuitive and align with known AKI pathophysiology. Crucially, these risk factors are modifiable. Importantly, Figure 3 highlights that while the same core physiologic domains drive predictions in both validation settings, their relative contributions shift between the Northeastern external cohort and the COVID-19 era temporal cohort in clinically interpretable ways. Across both cohorts, MAP dynamics and renal output remained dominant and consistent: MAP PSC and urine output were the top two contributors in both the external and temporal SHAP summaries, supporting that hemodynamic–renal coupling is a transportable signal for 24 h AKI risk despite shifts in geography and time. Where the cohorts diverged, the differences align with the known clinical context. In the temporal (COVID-19 era) cohort (Figure 3b), oxygenation dynamics appear more granular and prominent—beyond SpO_2_ PSC and longer-horizon SpO_2_ slope, a within-window SpO_2_ slope emerges among the top predictors only temporally—consistent with an era characterized by greater respiratory instability and oxygenation volatility (e.g., hypoxemic respiratory failure, frequent changes in ventilatory/oxygen support, and evolving ICU protocols). Clinically, such rapid oxygenation swings can plausibly interact with renal risk via hypoxemia-related systemic stress, inflammatory burden, and cardio-pulmonary–renal interactions that affect renal perfusion and venous congestion. Conversely, in the Northeastern external cohort (Figure 3a), proxies of respiratory drive and volume-management/therapy appear among the top predictors (e.g., respiratory-rate PSC and IV loop diuretics), which may reflect differences in case mix and bedside practice patterns across the participating hospitals—where tachypnea may capture physiologic stress and evolving respiratory compromise, and diuretic exposure may act as a marker for congestion/de-resuscitation strategies or underlying cardio-renal vulnerability that modulates AKI risk. Notably, the binary “shock” feature appears among the top predictors in the temporal cohort but not in the external cohort, suggesting that during the COVID-19 era, it carried incremental information beyond continuous MAP trends—potentially reflecting abrupt clinical deterioration phenotypes—whereas in the external cohort, shock-related risk may have been more fully captured by continuous hemodynamic and urine-output trajectories, reducing the marginal contribution of the binary indicator. We also observed a coherent shift in metabolic signal: the temporal cohort placed a relatively greater emphasis on the anion gap compared with the bicarbonate gap, whereas the external cohort showed the reverse. This pattern is compatible with cohort differences in prevailing acid–base phenotypes and measurement/ordering practices, which can make certain laboratory changes more informative (i.e., “new” to the model) in one setting than another. Taken together, these cohort-specific SHAP shifts are likely clinically meaningful reflections of case mix, severity, and practice-pattern differences rather than incidental noise, while the consistent directionality of the main effects (e.g., falling MAP/low urine output increasing risk; worsening oxygenation increasing risk) supports the robustness of the model’s physiologic reasoning across settings.

The SHAP importance analysis indicated, for example, that a downward trend in blood pressure was one of the strongest contributors to predicted AKI risk. This finding is actionable: clinicians can respond by optimizing hemodynamic (fluids and vasopressors) to improve renal perfusion. Similarly, low urine output and rising creatinine flagged by the model would prompt a search for reversible causes (e.g., relieving obstruction, stopping nephrotoxic drugs, or improving cardiac output). The transparency of our model’s reasoning allows it to function not as a “black box” but as a supportive tool that highlights high-risk patient trajectories and their likely causes. In day-to-day practice, these explanations can anchor focused discussions during ward rounds or multidisciplinary huddles, turning an abstract risk score into a concrete set of modifiable problems around the bed. This is essential for clinician trust in and uptake of any AI-based tool. We also demonstrated clinical utility through decision curve analysis. The net benefit curves showed that using the EWS to trigger interventions would achieve better outcomes than either intervening on all ICU patients or on none of them across a wide range of risk thresholds. For instance, at a threshold of 0.20, the model provides a ≈0.05 net benefit, corresponding to ~5 additional true-positive alerts per 100 evaluations, compared with treat-none. This positive net benefit persisted up to high-risk thresholds (~80%), indicating that the model retains clinical value even if one adopts a more conservative alert trigger. By specifying a threshold (we chose 12.5% during development to balance sensitivity and precision), we ensured the alert operates in a range that catches most impending AKI cases (recall ~75%) while keeping false positives at a manageable level. The high sensitivity at our chosen operating point means the system acts as an effective “sentry,” identifying three out of four patients who will develop AKI in the next 24 h—a substantial opportunity for early intervention. These results underscore that the model is not only statistically sound but also practically useful in guiding clinical decisions [37]. In this way, the EWS can function as a structured checklist for kidney-protective care at the bedside, complementing—but not replacing—clinical judgment and multidisciplinary discussion.

### 4.4. Clinical Implementation Pathway

Translating an early warning model into practice requires thoughtful integration into clinical workflows. We envision deploying this EWS directly within the electronic health record environment for real-time bedside use. A key advantage of our approach is that it can run on hospital premises using local EHR data with minimal computational power, thus avoiding privacy and regulatory barriers associated with sending data to third-party cloud services. By keeping the computation in-house, our score bypasses that barrier. This will enable other institutions to reproduce the model’s performance on their data or tailor the system to their environment, fostering transparency and collaboration. Hospitals can further calibrate the model using their own data for increased discrimination. However, before activating clinical alerts, this pathway requires prospective validation within the target EHR workflow and a staged rollout with explicit alert governance. We propose (i) shadow (silent) deployment in which the model runs in real time but does not notify clinicians, enabling prospective performance assessment, drift monitoring, data-latency checks, and refinement of thresholds; (ii) controlled activation in a limited pilot (e.g., bed-by-bed or unit-by-unit), coupling alerts to pre-defined response bundles and stop rules, with clearly specified recipients (e.g., the primary ICU team and bedside nurse), a fixed refresh cadence (e.g., every 6 h) but rate-limited notifications (e.g., no more than one alert per patient per shift unless escalation criteria are met), and operational rules for suppression (e.g., existing AKI, ongoing renal replacement therapy, comfort-focused care, or recent completion of a prevention bundle) versus escalation (e.g., persistent high-risk predictions or a rapid rise in predicted risk, prompting attending review and/or nephrology input). Alert fatigue should be mitigated and audited quantitatively using metrics such as alerts per patient/day, repeat-alert rate, acknowledgement/override rates, and time-to-bundle completion, with adaptive thresholding and stop rules if alert burden exceeds predefined limits, calibration drifts materially, or safety signals emerge. (iii) Stepwise scale-up to additional beds/units can occur once feasibility and safety criteria are met, with ongoing monitoring and evaluation against outcomes that extend beyond process measures (e.g., AKI incidence and stage progression, renal replacement therapy use, ICU/hospital length of stay, and mortality).

In practice, an alert from our model could prompt clinicians to institute nephroprotective measures: for example, postponing or avoiding contrast-enhanced imaging, when possible [24], optimizing volume status and perfusion pressure in line with sepsis or shock protocols [8], and reviewing medications for potential nephrotoxins (such as NSAIDs, vancomycin, or ACE inhibitors). By acting earlier, it may be feasible to attenuate or even prevent some kidney injuries that would otherwise fully manifest. Over time, systematic use of such an alert-and-response pathway has the potential to shift units from reacting to established AKI towards a culture of routine kidney risk surveillance, with implications for dialysis demand, ICU length of stay, and downstream chronic kidney disease.

Equally important is clinician engagement and ease of use. The utilization of SHAP values and variable importance in our pipeline allows a treating physician or bedside nurse to not only see that a patient is at (for example) 40% risk for AKI, but also to understand why—perhaps the mean blood pressure trend or a spike in vasopressor dose is contributing. Armed with that information, the care team can take proactive measures. The goal is to embed the EWS seamlessly into decision-making, so that it functions like a familiar clinical decision support tool rather than an external algorithm. As part of implementation, education and protocol development are critical. We propose a standardized response protocol when the EWS alert is triggered to ensure that risk information is acted upon consistently and effectively. In prior studies, AKI e-alerts alone (without guidance) did not significantly improve patient outcomes [39], likely because awareness alone is insufficient to change management. To address this, our implementation strategy couples the risk alert with a targeted “AKI prevention bundle.” This approach is supported by evidence that pairing alerts with specific recommendations yields better care processes and can reduce AKI severity; beyond process improvements, recent reviews emphasize that AKI evaluation and management are rapidly evolving—with increasing use of biomarkers to complement creatinine/urine-output monitoring and ongoing investigation of regenerative/targeted therapies; a future iteration of the EWS could incorporate such biomarker-informed risk stratification once validated for ICU implementation. A suggested protocol is present in the Supplementary Methods.

### 4.5. Ethical and Equity Considerations

Implementation of this EWS also raises important ethical considerations. Although our subgroup analyses did not reveal major performance gaps by sex or race, the model is trained on observational EHR data that inevitably reflect existing patterns of access, treatment, and documentation. If deployed uncritically, the system could therefore help perpetuate or even amplify pre-existing inequities—for example, by directing scarce nephrology or ICU resources preferentially towards groups who are already more likely to be monitored and have richer data recorded, while under-recognizing risk in patients whose care is fragmented or incompletely documented. Fairness monitoring should thus be an explicit part of implementation: institutions adopting the EWS should routinely audit calibration, false-negative rates, and intervention rates across key demographic and clinical subgroups, and be prepared to adjust thresholds or workflows if systematic disparities emerge. To support accountability, the model is designed and presented as an assistive tool rather than an autonomous decision-maker, and ultimate responsibility for treatment decisions remains with the clinical team. Local governance structures—such as ICU quality committees or ethics boards—should oversee how alerts are embedded into protocols, define acceptable performance and equity targets in advance, and periodically review both patient outcomes and potential unintended consequences. Transparency of the model, facilitated by SHAP-based explanations, helps clinicians understand why an alert is generated and provides a basis for contesting or overriding recommendations when they conflict with clinical judgment. At the same time, any alerting system carries a risk of alert fatigue: excessive or poorly targeted notifications may erode trust, lead to desensitization, and ultimately harm patients if important warnings are ignored. Selecting thresholds through decision-curve analysis, piloting the tool with explicit stop rules, and iteratively refining the alert policy in partnership with frontline staff are therefore crucial safeguards. More broadly, we advocate that institutions treat the EWS as part of a learning health system, with continuous surveillance for performance drift, equity impact, and workflow burden, and with clear mechanisms for pausing, recalibrating, or decommissioning the tool if its harms outweigh its benefits.

### 4.6. Strengths and Contributions of This Study

Our study has several strengths. It is one of the few AKI prediction efforts to undergo comprehensive external validation, including a challenging temporal validation during a pandemic. The model maintained excellent calibration after minor Platt scaling. We also prioritized interpretability and clinical relevance in model design, employing features that clinicians routinely monitor and that are widely available in different settings. These choices enhance the model’s credibility and ease of use in practice. Moreover, we adhered to TRIPOD and TRIPOD+AI reporting standards and conducted decision-curve and subgroup analyses, providing a thorough assessment of the model’s performance and utility. The cohort was carefully assembled to ensure clinical relevance: we excluded patients with pre-existing severe kidney dysfunction, those already on dialysis at ICU arrival, and those who developed AKI before the prediction window, focusing on truly incident AKI events. We avoided data leakage by using patient-level cross-validation (grouping all windows from the same patient in the same fold) and by censoring each ICU stay at the first AKI occurrence. Second, our model leverages domain knowledge through monotonic feature constraints—for example, it is constrained to predict higher risk with higher creatinine and lower risk with higher urine output, reflecting known physiology. These constraints, combined with gradient-boosted tree models (XGBoost) [31], yielded an accurate yet inherently more interpretable model. Fourth, we conducted extensive subgroup analyses, which showed consistent performance across patient demographics and comorbidities. Notably, the model did not exhibit major performance biases by sex or race—mitigating concerns that it would systematically underperform in minority groups. Taken together, our study’s methodological rigor and transparency set it apart from many previous AKI prediction models and provide confidence in the robustness of its findings.

### 4.7. Limitations and Future Directions

We acknowledge limitations as well. First, this was a retrospective study using observational EHR data; thus, intervention trials are needed to determine whether using the EWS to guide care can improve patient outcomes. We plan to recalibrate the model on retrospective data from our hospital and deploy the model prospectively. The associations we identified (e.g., hypotension leading to AKI) support face validity, but only a prospective implementation study can confirm causality—for example, showing that acting on an EWS alert prevents an AKI that would have occurred otherwise. Second, our model’s performance, while strong overall, was slightly lower in certain subgroups, like septic patients. It is possible that in conditions where AKI is often driven by complex, multi-factorial processes (e.g., septic shock), additional features or tailored models could further improve accuracy. Third, although we included two large, heterogeneous databases, both were based in the United States; the model may require recalibration or validation in non-US health systems, where baseline risks and practice patterns differ. We encourage international validations to ensure global applicability. Fourth, the model cannot be applicable in settings that lack equipment for a high percentage of features or patients who did not undergo creatine or urine-output testing. Moreover, our approach focused solely on tabular data; we will extend our next project to include image and text data. Lastly, practical issues such as alert fatigue and integration costs must be considered—if the alert threshold is set too low, it could overwhelm clinicians with false positives, whereas an overly high threshold might miss opportunities. We approached this by using decision-curve analysis to choose a sensible threshold, but real-world tuning will be important during deployment. Despite these limitations, our work provides a robust foundation for an AKI early warning system and takes important steps toward making such predictive tools clinically actionable.

## 5. Conclusions

For clinicians caring for critically ill adults, our findings suggest that an interpretable, routinely updated AKI early warning score can be used as a practical tool to identify patients who warrant intensified kidney-protective management before overt injury occurs. In conclusion, we developed a high-performing, interpretable AKI early warning model and validated it across centers and during a pandemic timeframe. The EWS offers substantial lead time for clinicians to intervene, and our results suggest that, if coupled with appropriate response protocols, it has the potential to improve patient outcomes. Future work will focus on prospective trials to test the EWS in live clinical settings and on integrating the tool within ICU workflows in a user-centered manner. By sharing our model and outlining an implementation strategy, we aim to accelerate the translation of this EWS into practice and contribute to reducing the burden of AKI through timely prevention. If prospective studies show that EWS-guided care reduces AKI incidence and severity, such systems could be incorporated into standard ICU care pathways and quality metrics, shifting AKI management from late recognition towards proactive prevention.

## Figures and Tables

**Table 1:** Prediction-window-level characteristics across the development, temporal validation, and external validation cohorts. Values are median (IQR) for continuous variables and n (%) for categorical variables; percentages use non-missing denominators within each cohort. Missing (%) indicates overall missingness for that variable in each cohort. ASMD is shown relative to the development cohort.

	Development		Temporal Validation			External Validation		
Characteristic	Median (IQR) or Count (%)	NA	Median (IQR) or Count (%)	NA	ASMD	Median (IQR) or Count (%)	NA	ASMD
Demographics								
Female sex	123,354 (45.9%)	0	7323 (47.9%)	0	0.06	7137 (45.0%)	0	0.03
Age (years)	66.0 (54.0, 76.0)	0	65.0 (54.0, 75.0)	0	0.07	63.0 (51.0, 74.0)	0	0.14
Length of stay (days)	3.6 (2.6, 6.0)	0	4.1 (2.8, 7.2)	0	0.19	3.8 (2.7, 6.2)	0	0.07
Black	28,837 (10.7%)	0	1188 (7.8%)	0	0.10	659 (4.2%)	0	0.26
Hispanic	11,398 (4.2%)	0	590 (3.9%)	0	0.02	150 (0.9%)	0	0.23
White	192,320 (71.5%)	0	7771 (50.9%)	0	0.28	14,181 (89.3%)	0	0.61
Asian	7441 (2.8%)	0	861 (5.6%)	0	0.02	152 (1.0%)	0	0.18
Other race	28,926 (10.8%)	0	4866 (31.9%)	0	0.39	735 (4.6%)	0	0.39
Cardiothoracic ICU	50,064 (18.6%)	0	2776 (18.2%)	0	0.09	1670 (10.5%)	0	0.45
Medical ICU	33,188 (12.3%)	0	1443 (9.4%)	0	0.21	530 (3.3%)	0	0.48
Surgical/Trauma ICU	44,240 (16.5%)	0	2465 (16.1%)	0	0.30	4136 (26.1%)	0	0.22
Neurology ICU	37,216 (13.8%)	0	7524 (49.3%)	0	0.68	4108 (25.9%)	0	0.38
Mixed ICU	104,214 (38.8%)	0	1068 (7.0%)	0	0.19	5433 (34.2%)	0	0.46
Height (cm)	170.2 (163.0, 178.0)	11.7	170.2 (163.0, 178.0)	35.8	0.08	170.0 (160.0, 177.8)	0.1	0.04
Weight (kg)	76.8 (64.8, 90.0)	2.1	77.9 (65.5, 92.0)	6.3	0.07	79.0 (65.1, 94.0)	1.3	0.12
Comorbidities								
AMI	26,614 (9.9%)	0	1113 (7.3%)	0	0.03	1450 (9.1%)	0	0.05
AHF	14,095 (5.2%)	0	1124 (7.4%)	0	0.04	1064 (6.7%)	0	0.21
Chronic heart failure	32,447 (12.1%)	0	1383 (9.1%)	0	0.07	1568 (9.9%)	0	0.07
Atrial fibrillation	43,088 (16.0%)	0	2833 (18.5%)	0	0.04	2347 (14.8%)	0	0.29
SVT	7167 (2.7%)	0	652 (4.3%)	0	0.17	388 (2.4%)	0	0.05
History of cardiac arrest	1476 (0.5%)	0	141 (0.9%)	0	0.06	108 (0.7%)	0	0.02
Hypertension	139,446 (51.9%)	0	9189 (60.2%)	0	0.06	7408 (46.7%)	0	0.24
COVID-19	-	-	1726 (11.3%)	0	-	-	-	-
COPD	46,089 (17.1%)	0	1607 (10.5%)	0	0.06	2509 (15.8%)	0	0.09
Chronic kidney disease	27,427 (10.2%)	0	771 (5.0%)	0	0.20	1645 (10.4%)	0	0.04
Malignant cancer	23,989 (8.9%)	0	2228 (14.6%)	0	0.01	1457 (9.2%)	0	0.13
Metastatic solid tumor	11,152 (4.1%)	0	978 (6.4%)	0	0.05	466 (2.9%)	0	0.18
Cirrhosis	8185 (3.0%)	0	360 (2.4%)	0	0.04	469 (3.0%)	0	0.04
Hematological cancer	5477 (2.0%)	0	446 (2.9%)	0	0.03	333 (2.1%)	0	0.07
Pulmonary embolism	8528 (3.2%)	0	701 (4.6%)	0	0.07	629 (4.0%)	0	0.01
Shock	54,466 (20.3%)	0	3778 (24.7%)	0	0.33	5790 (36.5%)	0	0.31
Ischemic stroke	19,752 (7.3%)	0	2061 (13.5%)	0	0.18	1370 (8.6%)	0	0.03
Diabetes	66,932 (24.9%)	0	3250 (21.3%)	0	0.02	2842 (17.9%)	0	0.12
Sepsis	46,700 (17.4%)	0	2110 (13.8%)	0	0.38	3053 (19.2%)	0	0.43
Life-threatening arrhythmias	12,483 (4.6%)	0	538 (3.5%)	0	0.01	792 (5.0%)	0	0.02
ICH/SAH	28,929 (10.8%)	0	3513 (23.0%)	0	0.18	2348 (14.8%)	0	0.02
Laboratory values								
Hemoglobin (g/dL)	10.5 (9.0, 12.1)	32.7	11.0 (9.2, 12.6)	34.9	0.19	10.6 (9.0, 12.3)	36.9	0.06
MCHC (g/dL)	33.2 (32.1, 34.2)	36.9	32.8 (31.8, 33.6)	35.6	0.38	33.1 (32.3, 33.9)	40.5	0.13
MCV (fL)	91.0 (87.0, 94.5)	36.9	91.5 (88.0, 95.5)	35.6	0.16	90.2 (86.7, 94.1)	40.5	0.02
Platelets (10^3^/uL)	186.5 (137.0, 250.0)	35.3	196.5 (142.2, 261.0)	35.5	0.05	184.0 (133.0, 246.0)	40.5	0.06
RDW (%)	14.2 (13.2, 15.6)	39.3	13.8 (13.0, 15.1)	35.7	0.22	14.6 (13.6, 16.3)	40.7	0.21
Creatinine (mg/dL)	0.8 (0.7, 1.1)	27.6	0.8 (0.7, 1.1)	30.9	0.05	0.9 (0.7, 1.2)	32	0.12
BUN (mg/dL)	16.0 (11.0, 24.0)	27.8	15.3 (11.0, 22.0)	30.9	0.09	18.0 (12.3, 28.0)	31.5	0.14
Glucose (mg/dL)	126.0 (105.0, 157.0)	17.0	129.0 (107.0, 158.0)	34.1	0.01	126.8 (107.9, 150.0)	10.2	0.13
WBC (10^3^/uL)	10.9 (8.0, 14.8)	34.9	11.0 (8.2, 14.8)	35.7	0.01	10.8 (8.0, 14.5)	40.7	0.01
Anion gap (mEq/L)	13.0 (11.0, 15.0)	39.7	11.0 (9.0, 13.0)	31.3	0.65	9.5 (7.0, 11.5)	43.9	0.78
Bicarbonate (mEq/L)	23.0 (21.0, 26.0)	34.1	23.0 (21.0, 25.0)	31.1	0.18	24.0 (21.5, 27.0)	32.2	0.19
Chloride (mEq/L)	105.0 (101.0, 108.5)	26.9	104.5 (101.0, 108.0)	31.0	0.12	105.0 (101.0, 109.0)	31.7	0.03
Sodium (mEq/L)	139.0 (136.0, 141.5)	25.9	138.5 (136.0, 141.0)	30.9	0.02	138.0 (135.7, 141.0)	30.0	0.02
Potassium (mEq/L)	4.0 (3.7, 4.4)	24.5	4.1 (3.8, 4.4)	30.9	0.09	4.0 (3.7, 4.3)	31.0	0.10
eGFR (mL/min/1.73 m^2^)	90.3 (64.9, 103.9)	27.6	91.7 (69.7, 104.0)	30.9	0.09	89.0 (59.8, 105.7)	32.0	0.03
Vital signs								
Heart rate (bpm)	83.3 (73.6, 95.6)	0.3	80.8 (71.6, 91.7)	0.1	0.18	85.8 (74.5, 97.9)	0	0.08
Respiratory rate (breaths/min)	18.5 (16.3, 21.4)	1.3	18.4 (16.3, 21.2)	1.6	0.02	18.4 (16.0, 21.3)	0.1	0.04
Temperature (C)	36.8 (36.6, 37.1)	4.9	36.8 (36.6, 37.1)	1.3	0.01	37.0 (36.6, 37.4)	0.3	0.21
SpO_2_ (%)	97.2 (95.7, 98.8)	0.5	96.9 (95.2, 98.4)	0.1	0.18	96.9 (95.3, 98.5)	1.1	0.16
Urine output (mL)	990.0 (650.0, 1475.0)	22.1	850.0 (550.0, 1300.0)	6.7	0.24	1025.0 (680.0, 1550.0)	3.1	0.03
Systolic BP (mmHg)	121.2 (109.1, 135.1)	0.3	115.9 (105.15, 128.8)	0.1	0.04	123.5 (112.4, 133.5)	0.1	0.07
Diastolic BP (mmHg)	65.6 (58.0, 74.8)	0.3	62.2 (54.8–71.0)	0.1	0.02	61.5 (52.4, 70.3)	0.1	0.02
Mean BP (mmHg)	78.6 (71.8, 87.4)	0.4	82.3 (74.9, 91.8)	0.1	0.30	79.3 (71.4, 88.1)	0.3	0.03
Invasive BP monitoring	73,384 (27.3%)	0	5998 (39.3%)	0	0.54	4054 (25.5%)	0	0.39
Treatment								
Loop diuretics (PO)	2244 (0.8%)	0	87 (0.6%)	0	0.06	147 (0.9%)	0	0.01
Loop diuretics (IV)	20,799 (7.7%)	0	1477 (9.7%)	0	0.14	1234 (7.8%)	0	0.09
Thiazides (PO)	2134 (0.8%)	0	108 (0.7%)	0	0.03	638 (4.0%)	0	0.20
Thrombolytics	922 (0.3%)	0	84 (0.5%)	0	0.03	160 (1.0%)	0	0.06
Insulin	39,830 (14.8%)	0	2960 (19.4%)	0	0.02	4488 (28.3%)	0	0.10
Non-insulin antidiabetics	2367 (0.9%)	0	35 (0.2%)	0	0.02	476 (3.0%)	0	0.24
Dopamine	3810 (1.4%)	0	14 (0.1%)	0	0.12	135 (0.9%)	0	0.01
Dobutamine	2834 (1.1%)	0	65 (0.4%)	0	0.02	39 (0.2%)	0	0.07
Norepinephrine	22,330 (8.3%)	0	1022 (6.7%)	0	0.19	1712 (10.8%)	0	0.09
Phenylephrine	9915 (3.7%)	0	1065 (7.0%)	0	0.02	1114 (7.0%)	0	0.39
Epinephrine	23,826 (8.9%)	0	1204 (7.9%)	0	0.17	1780 (11.2%)	0	0.04
Vasopressin	3048 (1.1%)	0	270 (1.8%)	0	0.10	292 (1.8%)	0	0.02
Milrinone	2042 (0.8%)	0	54 (0.4%)	0	0.09	131 (0.8%)	0	0.01
Statins	13,514 (5.0%)	0	1291 (8.5%)	0	0.12	1810 (11.4%)	0	0.03
ACEi/ARB	8935 (3.3%)	0	475 (3.1%)	0	0.08	1437 (9.1%)	0	0.27
Warfarin	3397 (1.3%)	0	56 (0.4%)	0	0.12	644 (4.1%)	0	0.19
Non-VKA anticoagulants	838 (0.3%)	0	77 (0.5%)	0	0.01	55 (0.3%)	0	0.06
CABG	4352 (1.6%)	0	538 (3.5%)	0	0.01	104 (0.7%)	0	0.31
Valve surgery	2990 (1.1%)	0	328 (2.1%)	0	0.09	113 (0.7%)	0	0.26
PCI	1257 (0.5%)	0	55 (0.4%)	0	0.09	63 (0.4%)	0	0.08
Contrast given	3476 (1.3%)	0	42 (0.3%)	0	0.21	43 (0.3%)	0	0.20
Outcome								
Acute kidney injury	32,049 (11.9%)	0	2617 (17.1%)	0	0.06	2250 (14.2%)	0	0.16

Abbreviations: AMI—acute myocardial infarction; AHF—acute heart failure; SVT—supraventricular tachycardia; COPD—chronic obstructive pulmonary disease; SAH—subarachnoid hemorrhage; ICU—intensive care unit; WBC—white blood cell (count); RDW—red cell distribution width; MCHC—mean corpuscular hemoglobin concentration; MCV—mean corpuscular volume; BUN—blood urea nitrogen; eGFR—estimated glomerular filtration rate; SpO_2_—peripheral oxygen saturation; BP—blood pressure; ACEi/ARB—angiotensin-converting enzyme inhibitor/angiotensin II receptor blocker; VKA—vitamin K antagonist; CABG—coronary artery bypass graft(ing); PCI—percutaneous coronary intervention; PO—by mouth (per os); IV—intravenous; ASMD—absolute standardized mean difference; NA—not available.

**Table 2:** Discrimination and calibration metrics for the final XGBoost model in development (10-fold cross-validation), temporal validation, and external validation cohorts. The 95% confidence intervals are from 2000 bootstrap resamples clustered by patient/stay.

Metric	Cohort		
Dataset/Metric	Development	Validation (Temporal)	Validation (External)
AUC	0.88 (0.84–0.90)	0.84 (0.80–0.87)	0.82 (0.81–0.84)
AUPRC	0.60 (0.57–0.62)	0.60 (0.56–0.64)	0.53 (0.50–0.57)
F1	0.57 (0.53–0.61)	0.58 (0.56–0.64)	0.58 (0.55–0.62)
F2	0.68 (0.60–0.70)	0.68 (0.62–0.73)	0.66 (0.61–0.68)
Recall	0.79 (0.75–0.86)	0.76 (0.73–0.80)	0.73 (0.67–0.78)
Precision	0.44 (0.41–0.52)	0.47 (0.43–0.54)	0.48 (0.42–0.54)
Specificity	0.84 (0.80–0.89)	0.79 (0.76–0.83)	0.86 (0.83–0.89)
Balanced accuracy	0.82 (0.78–0.86)	0.78 (0.76–0.83)	0.80 (0.78–0.84)
MCC	0.50 (0.46–0.55)	0.47 (0.43–0.49)	0.50 (0.44–0.54)
Brier score	0.07 (0.04–0.10)	0.10 (0.07–0.13)	0.09 (0.07–0.14)

Abbreviations: AUC—area under the curve; AUPRC—area under the precision-recall curve; MCC—Matthews correlation coefficient.

**Table 3:** Subgroup analyses on the temporal and external validation cohorts.

	Temporal Validation			External Validation		
Category	AUC	AUC Reference	*p*	AUC	AUC Reference	*p*
Age (50–74)	0.83 (0.82–0.85)	0.81 (0.78–0.84)	0.084	0.82 (0.80–0.83)	0.81 (0.79–0.84)	0.800
Age (≥75)	0.84 (0.82–0.86)	0.81 (0.78–0.84)	0.061	0.82 (0.80–0.84)	0.81 (0.79–0.84)	0.726
Race (Asian)	0.80 (0.78–0.84)	0.84 (0.83–0.86)	0.076	0.80 (0.67–0.93)	0.82 (0.81–0.83)	0.710
Race (Black)	0.81 (0.77–0.85)	0.84 (0.83–0.86)	0.142	0.82 (0.75–0.88)	0.82 (0.81–0.83)	0.902
Race (Hispanic)	0.80 (0.71–0.83)	0.84 (0.83–0.86)	0.097	0.66 (0.45–0.87)	0.82 (0.81–0.83)	0.140
Sex (Female)	0.84 (0.83–0.85)	0.83 (0.81–0.85)	0.372	0.82 (0.80–0.83)	0.82 (0.81–0.84)	0.560
Sepsis	0.79 (0.75–0.82)	0.83 (0.81–0.84)	0.004	0.79 (0.76–0.81)	0.82 (0.81–0.83)	0.030
Chronic kidney disease	0.86 (0.83–0.89)	0.84 (0.83–0.85)	0.145	0.80 (0.77–0.83)	0.82 (0.81–0.83)	0.181
Acute myocardial infarct	0.88 (0.85–0.90)	0.85 (0.82–0.88)	0.024	0.82 (0.79–0.85)	0.82 (0.81–0.83)	0.823
Acute heart failure	0.85 (0.83–0.88)	0.83 (0.82–0.84)	0.096	0.78 (0.74–0.82)	0.82 (0.81–0.83)	0.066
Diabetes (type 1 or 2)	0.84 (0.82–0.85)	0.84 (0.83–0.85)	0.973	0.81 (0.79–0.84)	0.82 (0.81–0.83)	0.614
Cirrhosis	0.86 (0.82–0.90)	0.84 (0.83–0.85)	0.321	0.79 (0.75–0.82)	0.82 (0.81–0.83)	0.046
Hypertension	0.84 (0.83–0.85)	0.83 (0.81–0.85)	0.256	0.83 (0.81–0.84)	0.81 (0.80–0.83)	0.324
Contrast	0.84 (0.71–0.97)	0.84 (0.83–0.85)	0.433	0.85 (0.81–0.88)	0.82 (0.81–0.83)	0.646
COVID-19	0.82 (0.79–0.84)	0.84 (0.83–0.85)	0.143	-	-	-

*p*-values are derived using DeLong’s test with Bonferroni correction. Reference groups were age <50 years, male sex, White race, and the absence of the specified condition/exposure (e.g., no sepsis, no CKD, no contrast, no COVID-19).

## Data Availability

The original data presented in the study are openly available in MIMIC-IV and eICU-CRD databases, at https://physionet.org for credentialed researchers who complete the required data use agreement. Code for development and evaluation, along with model weights, are accessible upon request.

## Supplementary Materials

The following supporting information can be downloaded at https://www.mdpi.com/article/10.3390/jcm15031191/s1, Figure S1. Study Cohort Selection and Data Exclusion Flowchart from eICU and MIMIC-IV Databases; Figure S2. Calibration curves for the final model in external validation (upper panel) and temporal validation (lower panel); Figure S3. Decision curve analysis for external validation (upper panel) and temporal validation (lower panel); Figure S4. Gain-based feature importance from the final XGBoost model (top 20 predictors); Figure S5. Hospital-level discrimination in the external eICU cohort; Figure S6. Event-level lead-time detection for temporal and external validation cohorts; Table S1. Hyperparameters in XGBoost grid search; Table S2. Monotonic Constraints; Table S3. Comparison of discrimination between unconstrained and monotonic XGBoost models; Table S4; Final predictor set used in the early warning score; Table S5. Discrimination by overall feature missingness in the prediction window; Table S6. Discrimination by AKI definition in temporal and external validation cohorts. AUC and AUPRC are reported for urine output (UO)-based and creatinine (SCr)-based AKI labels; Table S7. Alert burden metrics at the prespecified alert threshold (0.125) in temporal (MIMIC-IV) and external (eICU) validation cohorts. Rates are reported per patient-day and per 12-h shift; time-under-alert is length-of-stay capped; percentages refer to alert-level or stay-level proportions as labeled; Table S8. Distribution of false-positive alerts by time to the next AKI event in temporal and external validation cohorts; Table S9. Threshold-based error rates by subgroup at the prespecified alert threshold.

## Abbreviations

The following abbreviations are used in this manuscript:

0by25	International Society of Nephrology (ISN) “0by25” initiative (AKI prevention/management focus)
ACEi/ARB	angiotensin-converting enzyme inhibitor/angiotensin II receptor blocker
AHF	acute heart failure
AI	artificial intelligence
AKI	acute kidney injury
AMI	acute myocardial infarction
ASMD	absolute standardized mean difference
AUC	area under the curve
AUPRC	area under the precision–recall curve
BP	blood pressure
bpm	beats per minute
BUN	blood urea nitrogen
CABG	coronary artery bypass graft(ing)
CI	confidence interval
CITL	calibration-in-the-large
CKD	chronic kidney disease
cm	centimeter(s)
COPD	chronic obstructive pulmonary disease
COVID-19	coronavirus disease 2019
CRD	Collaborative Research Database (as in eICU-CRD)
DCA	decision curve analysis
ECE	expected calibration error
EHR	electronic health record
eGFR	estimated glomerular filtration rate
eICU-CRD	Electronic Intensive Care Unit Collaborative Research Database
EWS	early warning score
F1	F1-score (F-measure with β = 1)
F2	F2-score (F-measure with β = 2; weights recall more)
fL	femtolitre(s)
g/dL	grams per deciliter
h	hour(s)
HIPAA	Health Insurance Portability and Accountability Act
ICU	intensive care unit
ICH	intracerebral hemorrhage (as used in ICH/SAH)
IQR	interquartile range
IRB	institutional review board
ISN	International Society of Nephrology
IV	intravenous
KDIGO	Kidney Disease: Improving Global Outcomes
kg	kilogram(s)
MAP	mean arterial pressure
MCC	Matthews correlation coefficient
MCHC	mean corpuscular hemoglobin concentration
MCV	mean corpuscular volume
mEq/L	milliequivalents per liter
mg/dL	milligrams per deciliter
MI	myocardial infarction
min	minute(s)
mL	milliliter(s)
mL/kg/h (or mL/kg/hour)	milliliters per kilogram per hour
mL/min/1.73m^2^	milliliters per minute per 1.73 square meters (BSA-standardized)
MIMIC-IV	Medical Information Mart for Intensive Care IV
mmHg	millimeters of mercury
ML	machine learning
NA	not available
NHS	National Health Service
NSAIDs	nonsteroidal anti-inflammatory drugs
*p*	*p*-value
PCI	percutaneous coronary intervention
PO	per os (by mouth)
PROBAST	Prediction model Risk of Bias ASsessment Tool
PSC	Piecewise slope change
RDW	red cell distribution width
SAH	subarachnoid hemorrhage
SCr	serum creatinine
SD	standard deviation
SHAP	SHapley Additive exPlanations
SpO_2_	peripheral oxygen saturation
SVT	supraventricular tachycardia
TRIPOD	Transparent Reporting of a Multivariable Prediction Model for Individual Prognosis or Diagnosis
TRIPOD+AI	TRIPOD extension/guidance for AI/ML prediction models
UO	urine output
U.S./US	United States
VKA	vitamin K antagonist
WBC	white blood cell (count)
XGBoost	eXtreme Gradient Boosting
