# Supplementary material for: Forecasting ICU Acute Kidney Injury with Actionable Lead Time Using Interpretable Machine Learning: Development and Multi-Center Validation
Source: J Clin Med. 2026 Feb 3;15(3):1191. doi: 10.3390/jcm15031191 (PMC12898506; doi:10.3390/jcm15031191)
Supplement: Supplementary file 1 [file jcm-15-01191-s001.zip › jcm-4085537-supplementary.pdf]

Supplementary Methods

Data Sources and Cohort Assembly

This retrospective cohort study used two large critical care databases: the Medical Information Mart for Intensive Care (MIMIC-IV) and the Electronic Intensive Care Unit Collaborative Research Database (eICU-CRD). Both registries integrate hospital-wide electronic health records with ICU clinical information systems, providing a high-resolution longitudinal record for each ICU stay and capturing the dynamic evolution of patients' clinical status over time. Data in both sources are de-identified and routinely collected in the course of clinical care, which reduces the risk of selection bias associated with disease- or intervention-specific registries and reflects real-world practice patterns. For each eligible stay, we obtained detailed information on demographics, vital signs, laboratory measurements, medication exposures, and procedural data, as well as admission characteristics and ICU type where available. MIMIC-IV encompasses 94,458 ICU stays from Beth Israel Deaconess Medical Center with admission dates between 2008 and 2022, representing a single tertiary academic center with relatively standardized documentation and care pathways. In contrast, eICU-CRD captures 200,859 ICU stays from 208 hospitals across the United States during 2014–2015, spanning a diverse mix of community and academic institutions and a broad range of ICU types, staffing models, and documentation practices. This contrast between a deeply characterized single-center cohort and a heterogeneous multi-center cohort enabled us to evaluate model performance and robustness across distinct healthcare settings. Together, these datasets allowed us to study acute kidney injury (AKI) in both a single-center and a multi-center setting using a consistent data-extraction framework. Minimum sample size for model development was determined using the Riley et al. approach for binary prediction models (as implemented in pmsampsize in R), targeting a global shrinkage factor ≥0.90 and small optimism in model fit. Expected discrimination was set to AUC=0.80 based on published AKI prediction models in ICU settings. With an anticipated AKI incidence of 20% and ~30 candidate parameters, the minimum required development sample was ~1,320 ICU stays (≈264 AKI events), and since ML models are data hungry, we ensured that our development and evaluation cohorts substantially exceeded these minimum requirements in both total ICU stays and number of AKI events. The study was not pre-registered; and there was no public involvement in the design, conduction, reporting, interpretation, and dissemination of the current work.

We constructed a rolling-window cohort from all ICU stays that satisfied prespecified inclusion criteria. Each ICU stay was divided into a sequence of predefined prediction windows according to the framework described in the main Methods, so that the evolving risk of AKI could be assessed repeatedly over time during the ICU stay. Because the eICU database aggregates data from multiple hospitals with heterogeneous documentation practices, we first restricted the eICU cohort to hospitals with adequate coverage of key clinical data domains. Using the hospital-level coverage table, we evaluated structured coverage of diagnosis and past medical history fields, laboratory results, nursing charting, treatments and procedures, intake and output, medication and infusion records, and respiratory charting. Hospitals were flagged for exclusion if any of these domains were consistently labeled as having "none" or "low" coverage, or if combinations of diagnosis- and medication-related domains (e.g., admission diagnoses, medication orders, infusion drugs) were simultaneously poorly covered. A set of seven prespecified coverage rules, based on these domain-level flags, was used to identify and remove hospitals where missingness largely reflected systematic under-documentation rather than true absence of measurements. This hospital-level pruning step was performed before constructing patient-level rolling windows, ensuring that the remaining eICU sites provided sufficiently rich and internally consistent data to support dynamic AKI risk modelling. Out of 208 hospitals, 134 hospitals were used in our model development. Cohort selection and exclusion followed the same steps and order on both datasets (Figure S1), and is consistently similar to that done in previous reliable models. To ensure accurate data extraction and cohort construction, we first excluded 74 eICU centres in which dialysis and nursing charting were incompletely or inconsistently recorded, thereby avoiding the use of unreliable data in model development and evaluation. Further, to guarantee that each ICU stay contributed at most one outcome event, we censored follow-up at the first incident AKI and discarded all subsequent windows from that stay. We further excluded ICU stays in which kidney replacement therapy was already in use at the time of ICU entry, as these patients had established severe kidney dysfunction at baseline. Stays in which AKI had already developed by the first prediction window were also removed to focus the analysis on incident AKI. In addition, we excluded stays with documented end-stage kidney disease and those with missing baseline AKI status. Adults aged 18–88 years at ICU admission were eligible for inclusion; entries recorded as ">89" years were excluded because ages above this threshold are masked in the source data for privacy, which precludes accurate age-based characterization. Further, we excluded ICU stays with a length of stay <2 days, as very short admissions both provide insufficient longitudinal data for constructing prediction windows and represent transient ICU encounters with limited opportunity for incident AKI to develop or to be captured by laboratory data. Patients with a history of solid-organ transplantation and pregnant patients were excluded, as these groups have notably distinct immunologic, hemodynamic, and pharmacologic profiles, leading to AKI risk trajectories and management strategies that differ substantially from the general ICU population and could compromise the clinical interpretability and generalizability of the model. We further dropped entire stays where we saw inconsistent results such as weight 700kg or heart rate 400. Finally, we dropped stays, where rows missing more than 50% predictors or any of age, sex, or race were missing. We further excluded windows that contained physiologically implausible measurements (e.g., body weight 700 kg or heart rate 400 beats/min), as these values likely reflected data-entry errors. Finally, we removed stays in which more than 50% of candidate predictors were missing or any key demographic variable (age, sex, or race) was unavailable, to minimize bias from sparsely characterized records and ensure reliable model training and validation.

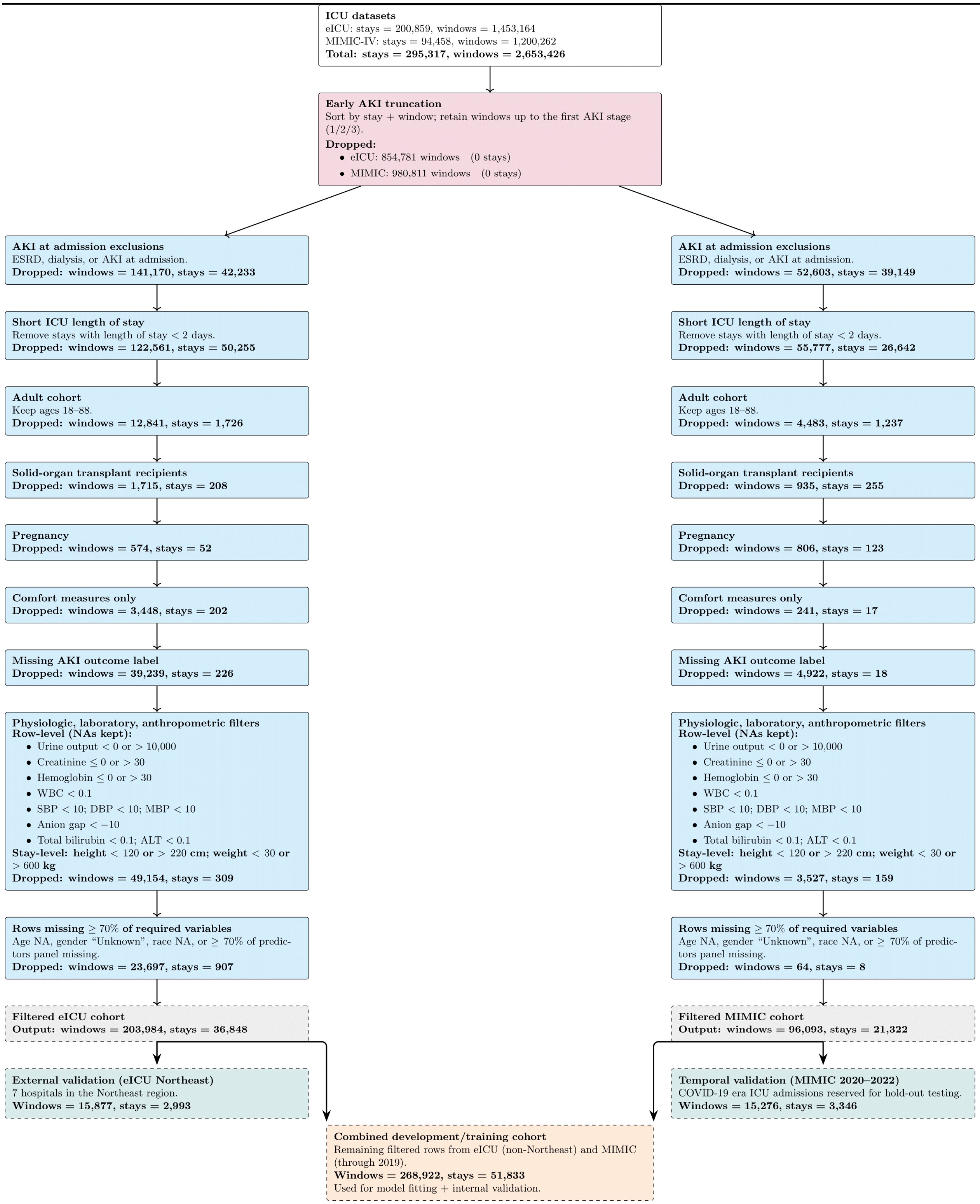

Figure S1. Study Cohort Selection and Data Exclusion Flowchart from eICU and MIMIC-IV Databases.

After applying these criteria, we defined two held-out test frames to evaluate temporal and geographic generalization. For temporal generalization, we created a MIMIC-IV test cohort comprising ICU stays with admission dates from 2020 to 2022, a period that overlaps with the COVID-19 pandemic, yielding 3,346 ICU stays and 15,278 prediction windows. For geographic generalization, we derived an eICU test cohort by restricting ICU stays to hospitals located in the U.S. Northeast region (7 hospitals), resulting in 2,993 ICU stays and 15,877 prediction windows. All remaining eligible ICU stays from MIMIC-IV and eICU-CRD that were not allocated to either held-out

test frame were pooled to form the training dataset, comprising 268,922 windows from 51,833 distinct stays. Internal validation within this training dataset was performed using 10-fold cross-validation. Unique patients (rather than individual stays or windows) were randomly assigned to one of ten folds, and at each iteration models were fitted on nine folds and evaluated on the remaining fold; the same folds were used across model variants to ensure comparability. To prevent information leakage across data partitions, all prediction windows from a given patient—including, when applicable, windows arising from multiple ICU stays for the same individual—were assigned to the same cross-validation fold and to the same train–test partition.

Rolling-Window Prediction Framework and Outcome Definition

Each ICU stay was decomposed into a sequence of overlapping prediction windows, each defined by a 12-hour feature (look-back) window, followed by a 2-hour gap to reduce label leakage, and a 24-hour outcome horizon. Operationally, once at least 12 hours of data were available for a given ICU stay, we constructed prediction windows that were updated every 6 hours. For a prediction time t, model inputs were derived from the preceding 12 hours of data (from t − 12 h to t), no information from the subsequent 2-hour gap (t to t + 2 h) was used, and the outcome was evaluated within the following 24 hours (t + 2 h to t + 26 h). The 6-hour stride thus creates partially overlapping windows while limiting redundancy, and ensures at most two risk updates per patient during a standard 12-hour shift, aligning with typical ICU rounding and handoff practices. We empirically compared several combinations of feature-window length, gap duration, and outcome horizon and selected the 12 h / 2 h / 24 h configuration because it best matched the temporal resolution and routine measurement frequency of the available variables in the training data, while providing a clinically meaningful balance between timely situational awareness and alert burden. Targeting a 24-hour horizon was intended to surface elevated risk sufficiently early to allow preventive and kidney-protective interventions, consistent with KDIGO's focus on short-term AKI evolution and dynamics.

The primary endpoint was binary **incident AKI** (KDIGO stages 1–3) occurring **after** the prediction cutoff and **within** the subsequent 24-hour outcome horizon. For each prediction time t(the end of the 12-hour feature window), we defined a leakage-safe label

$$y(t) = \mathbb{1}\{\tau \in (t + 2\,\mathrm{h}, t + 26\,\mathrm{h}]\},$$

where $\tau$is the **AKI onset time**. We constructed a time-indexed KDIGO staging process $S(u)$over each ICU stay and set

$$\tau = \inf\{u: S(u) \geq 1\}, S(u) = \max\{S_{\mathrm{SCr}}(u), S_{\mathrm{UO}}(u), S_{\mathrm{RRT}}(u)\},$$

evaluated only using data available strictly **before** each assessment timestamp u. **Creatinine-based staging** was computed at each serum creatinine timestamp $u$using time-varying baselines derived solely from prior values:

$$b_{48}(u) = \min_{v \in [u-48\mathrm{h}, u)} \mathrm{SCr}(v), b_{7d}(u) = \min_{v \in [u-7\mathrm{d}, u)} \mathrm{SCr}(v).$$

Stage 1 at time $u$was assigned if $\mathrm{SCr}(u) \geq b_{48}(u) + 0.3$ mg/dL(acute absolute rise within 48h) or $\mathrm{SCr}(u) \geq 1.5\ b_{7d}(u)$(relative rise over 7 days); stage 2 if $\mathrm{SCr}(u) \geq 2\ b_{7d}(u)$; and stage 3 if $\mathrm{SCr}(u) \geq 3\ b_{7d}(u)$, or if $\mathrm{SCr}(u) \geq 4.0$ mg/dLwith an associated acute increase as operationalized in the MIMIC-IV KDIGO concept implementation. When $b_{48}(u)$and/or $b_{7d}(u)$were undefined (no prior creatinine in the lookback windows), we did not impute any baseline from future measurements or long-horizon history; the creatinine criterion at $u$was treated as unavailable by construction, preventing label leakage. Urine-output staging was computed at hour-aligned timestamps $u$by aggregating charted urine volumes into hourly totals and forming rolling, weight-normalized rates

$$r_k(u) = \frac{V(u-k, u]}{w(u)\ \Delta_k(u)} (\mathrm{mL/kg/h}), k \in \{6,12,24\},$$

where $V(u-k, u]$is the cumulative urine volume in the preceding $k$hours, $w(u)$is contemporaneous body weight (kg), and $\Delta_k(u)$is the observed documentation span used for that rolling calculation (to avoid assuming fully observed windows when charting is sparse). Following the concept code, urine-output staging was not assigned until the patient had been in the ICU for at least 6 hours, and we required sufficient documentation coverage ($\Delta_6(u) \geq 2$ h, $\Delta_{12}(u) \geq 5$ h, $\Delta_{24}(u) \geq 11$ h) before applying thresholds. Stage 1 at u was assigned if $r_6(u) < 0.5$with $\Delta_6(u) \geq 2$ h; stage 2 if $r_{12}(u) < 0.5$with $\Delta_{12}(u) \geq 5$ h; and stage 3 if $r_{24}(u) < 0.3$with $\Delta_{24}(u) \geq 11$ h, or if anuria occurred ($r_{12}(u) = 0$ with $\Delta_{12}(u) \geq 5$ h). Body weight $w(u)$was derived from ICU charted **admission** and **daily** weights (Admit Wt and Daily Weight) and represented as a piecewise-constant series with explicit start/stop times: the first admission weight was anchored to ICU admission with a 2-hour timing tolerance, each subsequent weight applied from its start time until the next recorded weight (or ICU discharge, plus a 2-hour tolerance), and initial gaps were backfilled by carrying the first available weight backward to ICU admission (again with the same tolerance). If no weight was available at u, urine-output staging was treated as missing. **RRT** was treated as KDIGO stage 3 from the documented initiation time onward. To ensure the endpoint reflected **incident** (not prevalent) AKI, we excluded ICU stays meeting any KDIGO criterion before the first eligible prediction cutoff and censored all subsequent prediction windows after τ; additionally, we will report the relative contribution of creatinine-, urine-output-, and RRT-based criteria to AKI onset classification.

Feature Selection and Processing

All preprocessing and feature engineering steps were performed strictly within the training portion of each cross-validation split. For every fold, all transformations (including calculation of summary statistics, encoding schemes, and variable filtering) were learned exclusively on the training data and then applied unchanged to the corresponding validation and test sets. This approach was chosen to prevent data leakage and to ensure that performance estimates reflected a realistic, prospective deployment scenario. Candidate predictors were

identified a priori from the nephrology and critical-care literature and encompassed a broad range of clinically relevant domains: demographic characteristics, chronic comorbid conditions, vital signs, laboratory measurements, medication exposures, ventilatory support variables, and procedural indicators. These variables were extracted from the electronic health record and organised at the patient level. For variables recorded as repeated measurements over time, continuous time series were summarised over the predefined observation window using several simple yet informative statistics, including the average value, standard deviation, and linear slope. In addition to within-window summaries, we incorporated out-of-window trend features that extend prior to the 12-hour prediction window, specifically 24-hour and 48-hour slopes as well as a piecewise slope metric. The primary motivation for these features is to capture longer-horizon trajectories that may be clinically informative but not fully observable from the current short window alone. Many pathophysiological processes in critically ill patients—such as evolving renal dysfunction, inflammatory escalation, hemodynamic deterioration, or delayed treatment response—manifest as gradual changes over one to two days rather than abrupt shifts confined to a single 12-hour segment. Longitudinal slopes computed over 24–48 hours therefore provide a more stable estimate of directionality (improving vs. worsening) and reduce sensitivity to transient noise, sporadic measurement timing, or short-lived fluctuations. The piecewise slope was defined as the difference between the current-window slope and the slope estimated over the preceding 24 hours (i.e., current slope − prior 24-hour slope). This feature is intended to quantify acceleration or deceleration in the trajectory—distinguishing patients with steadily worsening trends from those whose deterioration is newly emerging or rapidly intensifying. In clinical terms, this can reflect meaningful shifts such as the onset of acute kidney injury after a period of stability, loss of response to therapy, or abrupt worsening of perfusion. Because these extended-history features require prior observations, they are structurally unavailable in the earliest windows of a patient stay. Accordingly, the 24-hour and 48-hour slope-based variables (and the piecewise slope) are expected to be undefined—and therefore recorded as NULL—for initial windows (e.g., the first to third windows), where insufficient historical data exist to support their computation. This is not treated as a data-quality defect but as a deterministic consequence of feature definition and temporal alignment. Importantly, the model is not permitted to "peek" into future measurements to populate these features; leaving them NULL in early windows preserves temporal integrity and prevents inadvertent leakage. To ensure the model can appropriately interpret this structured absence of information, we additionally included a dedicated missingness indicator column for these out-of-window trend features (and only for these features). This indicator explicitly flags whether the extended-history trend was computable at that time point, allowing the learning algorithm to differentiate between (i) early-window unavailability due to insufficient prior history and (ii) observed values later in the stay. Restricting missingness indicators to this specific feature subset avoids unnecessary dimensional expansion and limits the risk of introducing spurious patterns from broadly encoding missingness across all variables.

Categorical predictors (e.g., sex, admission type, medication classes, and procedure indicators) were encoded using one-hot encoding, producing binary indicator variables for each level to facilitate direct use in downstream machine learning models without imposing an artificial ordinal structure. Data for both cohorts were extracted using harmonized SQL pipelines developed in accordance with the official documentation and guidebooks supplied by the respective data providers. Demographic characteristics were obtained from admission-level tables and included age, sex, and race. All patients in the final selected cohort had age and sex, for some race was list was listed as others or unknown, which we categorised in their own category; importantly, the dataset didn't have major differences in race categories, as both come from the US, and both were preprocessed by the same team. Chronic comorbidities were identified using internationally standardized ICD-9 and ICD-10 diagnostic codes and operationalized such that, once documented, the corresponding comorbidity indicator was set to 1 from the diagnosis timestamp onward and retained for all subsequent windows. In contrast, acute conditions—specifically sepsis, shock, and intracranial hemorrhage/subarachnoid hemorrhage (ICH/SAH)—were not ascertained via ICD coding; instead, they were identified using prespecified, timestamped signals drawn from multiple clinical data sources (e.g., procedure events, laboratory results, vital signs, and microbiology tables), consistent with the data availability and structure of each database. Procedures were extracted using procedural ICD-9 and ICD-10 codes and encoded at the window level, with indicators set to 1 only when the procedure occurred within the corresponding 12-hour feature-extraction interval. Laboratory tests were retrieved using provider-specified item identifiers to ensure consistent test mapping across database schemas. Vital signs were derived from nurse-validated charting rather than direct device feeds, prioritizing clinically confirmed measurements and reducing the influence of high-frequency device artifacts and recording noise; this choice also supports broader deployability in settings without uniform bedside device integration or mature interoperability infrastructure. Finally, to prevent data leakage, all extractions and derived features were strictly constrained by timestamps, ensuring that each predictor was computed solely from observations occurring within its designated window. Missing values were not imputed. Instead, we leveraged the ability of tree-based ensemble models to natively handle missingness during the splitting process. In this setting, missingness is treated as an informative attribute rather than noise to be "corrected." Preserving the original sparsity structure of EHR time series enables the model to exploit clinically meaningful patterns in test ordering and availability—for example, laboratory tests that are obtained predominantly in sicker patients, or variables that are measured only under specific clinical concerns. Conventional imputation strategies could attenuate or obscure these patterns; therefore, we intentionally allowed missingness to remain as part of the feature space. Predictors with more than 50% missingness were excluded a priori, as such extensive sparsity was considered unlikely to yield stable or generalisable signal. In addition, predictors exhibiting near-zero variance (i.e. almost no variability across patients) were removed to reduce redundancy and improve numerical stability. To assess potential collinearity between predictors, we computed both Pearson and Spearman correlation coefficients, depending on the distributional characteristics and scale of the variables. These correlation analyses were used to characterise the dependence structure of the feature set and to ensure that highly correlated variables were appropriately recognised during subsequent modelling and interpretation.

Model Development, Sensitivity Analysis and Performance Evaluation

We trained gradient-boosted decision tree models using the XGBoost framework. XGBoost is well suited to heterogeneous EHR tabular data because it natively handles missing values by learning an optimal default direction for missingness at each split, provides stable performance across a wide range of data partitions, and is designed specifically for high-dimensional tabular inputs rather than images or text. In addition, XGBoost (i) captures complex non-linear effects and higher-order interactions through ensembles of shallow trees, (ii) incorporates multiple regularisation mechanisms (L1/L2 penalties, shrinkage, and row/column subsampling) that reduce overfitting, (iii) scales efficiently to large datasets with imbalanced outcomes, and (iv) supports monotonic constraints on selected predictors, which is advantageous when prior clinical knowledge suggests a directional effect. Hyperparameters were tuned using a random search over a predefined grid applied to the training portion of each cross-validation split. The grid varied the number of trees, tree depth, learning rate, row and column subsampling fractions, minimum number of observations per leaf, L1 and L2 penalties, and the minimum loss reduction required to create a new split; the full set of candidate values for each parameter is summarized in Table S2. For each sampled hyperparameter configuration, boosting iterations were subject to early stopping based on performance on an internal validation subset of the training fold, such that tree growth was halted once additional boosting rounds no longer improved validation performance. This procedure effectively limited overfitting, selected the optimal number of trees for each configuration, and allowed the final models to adapt their complexity to the signal present in the data. Because incident AKI windows were substantially less frequent than non-AKI windows, we used weighting rather than resampling to address class imbalance. First, we assigned a patient-level weight so that individuals with many windows (e.g. long or repeated ICU stays) did not disproportionately influence the loss function; this weight was then propagated to all windows arising from that patient. On top of this, we applied outcome-class weights at the window level to upweight AKI events. We empirically compared several AKI:non-AKI weighting schemes (including 1:1, 2:1, 3:1 and higher ratios) and found that a 5:1 ratio provided the best compromise between improved sensitivity for AKI, preservation of specificity, and stable calibration in cross-validation. In the final models, windows with an incident AKI outcome were therefore given five times the outcome-class weight of non-AKI windows, with the effective observation-level weight defined as the product of the patient-level and outcome-class components. The same weighting strategy was used consistently during cross-validation and when refitting the models on the full training set prior to evaluation in the two held-out test frames.

**Hyperparameter**	**Options**
Number of trees	c(100, 150, 300, 500, 600)
Max depth	3:10 by 1
Learn rate	c(0.01, 0.03, 0.05, 0.005)
Sample rate	0.6:0.9 by 0.1
Column sample rate	0.5:0.9 by 0.1
Column sample rate per tree	0.5:0.9 by 0.1
Min rows	c(1, 5, 10, 20, 50)
Lambda	c(0, 0.1, 1, 5, 10)
Alpha	c(0, 0.1, 1, 5, 10)
Min split improvement.	c(0, 1e-4, 1e-3)

Table S1. Hyperparameters in XGBoost grid search

We used ten-fold cross-validation at the stay level, such that all windows belonging to the same hospital stay were assigned to the same fold. This grouping preserved the natural clustering of repeated windows within stays and prevented information leakage between training and validation sets. Cross-validation folds were therefore defined on clusters (patients) rather than individual windows, in accordance with

TRIPOD+AI recommendations for clustered data (Supplementary file 2). Noteworthy, is we did 10-fold cross-validation here due to compute restraints. Preferably, each hospital should reside in a cluster; however, that would multiply the compute by 135x.
Because several predictors were expected a priori to have monotonic relationships with AKI risk, we fit two versions of the XGBoost model: one without monotonic constraints and one with monotonic constraints imposed on a prespecified subset of predictors. In the constrained model, the direction of monotonicity (increasing or decreasing risk with higher predictor values) was specified based on clinical reasoning, such that, for example, higher serum creatinine and blood urea nitrogen were constrained to increase risk, whereas higher urine output and blood pressure were constrained to decrease risk; the full list of constrained variables and their directions is provided in Table S3. Both the constrained and unconstrained models used the same feature set, weighting scheme, hyperparameter tuning grid, and cross-validation procedure described above. We compared the discrimination of the constrained and unconstrained models using DeLong's test for correlated AUCs on both the temporal (MIMIC-IV) and external geographic (eICU) test sets (Table S4). The estimated AUCs were nearly identical in both settings and neither comparison reached statistical significance. These findings indicate that introducing clinically motivated monotonic constraints did not materially alter model discrimination, while preserving the advantage of enforcing plausibly directional effects for selected clinical variables.

Clinical Variable	Monotonicity Direction
Serum creatinine	Increasing ↑
Urine output	Decreasing ↓
Systolic blood pressure	Decreasing ↓
Diastolic blood pressure	Decreasing ↓
Mean blood pressure	Decreasing ↓
Age	Increasing ↑
Blood urea nitrogen	Increasing ↑

Table S2. Monotonic Constraints.

Cohort	AUC	AUC monotone	Delong's P
Temporal	0.837	0.839	0.274
External	0.820	0.820	0.978

Table S3. Comparison of discrimination between unconstrained and monotonic XGBoost models.

To reduce the number of needed features in inference, we calculated SHAP during cross validation of the final model, and used mean absolute SHAP value to select the top 61 features. Difference in AUC and AUPRC between both models was marginal and insignificant so we decided to report the performance of the model with 61 features and validate it against our test datasets. The final set of 61 predictors spanned chronic comorbidities, demographics and anthropometrics, acute diagnoses, laboratory markers, vital-sign dynamics, and ICU therapies and procedures, as summarized in Table S1.

Demographics & anthropometrics	Chronic comorbidities	Laboratory markers	Vital signs & dynamics	Therapies, procedures & monitoring
Sex (male vs female)	Chronic heart failure	48-hour slope of haemoglobin concentration	48-hour slope of heart rate	Intravenous loop diuretic administration
Age at ICU admission (years)	Atrial fibrillation	Mean corpuscular haemoglobin concentration (MCHC), average	Piecewise change in heart rate slope	Thrombolytic therapy
Height at hospital admission (cm)	Hypertension	MCHC, within-window standard deviation	Respiratory rate, average	Insulin therapy
Weight (kg)	Chronic kidney disease	72-hour slope of mean corpuscular volume (MCV)	48-hour slope of respiratory rate	Dopamine infusion
	History of malignant cancer	Serum creatinine, average	Piecewise change in respiratory rate slope	Dobutamine infusion
	Diabetes mellitus	72-hour slope of serum creatinine	Body temperature (°C), average	Norepinephrine infusion

Demographics & anthropometrics	Chronic comorbidities	Laboratory markers	Vital signs & dynamics	Therapies, procedures & monitoring
	**Acute diagnoses / severity**	Blood urea nitrogen (BUN), average	48-hour slope of body temperature (°C)	Phenylephrine infusion
	Shock	72-hour slope of blood glucose	Piecewise change in body temperature slope	Epinephrine infusion
	Sepsis	White blood cell count, average	Peripheral oxygen saturation (SpO_2), average	Vasopressin infusion
	Intracerebral or subarachnoid haemorrhage	72-hour slope of white blood cell count	Slope of SpO_2 within the prediction window	Milrinone infusion
		Anion gap, average	48-hour slope of SpO_2	Coronary artery bypass graft (CABG) surgery
		Serum bicarbonate, average	Piecewise change in SpO_2 slope	Cardiac valve surgery
		48-hour slope of serum bicarbonate	Mean arterial blood pressure (MAP), average (combined)	Percutaneous coronary intervention (PCI)
		Serum chloride, average	48-hour slope of MAP (combined)	Urine output during the observation window
		48-hour slope of serum sodium	Piecewise change in MAP slope (combined)	Invasive arterial blood pressure monitoring performed
		48-hour slope of serum potassium		Intravenous iodinated contrast administered
		Estimated glomerular filtration rate, CKD-EPI 2021 (eGFR)		

Table S4. Final predictor set used in the early warning score.

We evaluate our model performance on different missingness percentages to assess whether models performance decays with lower or higher missingness rates (Table S5). Across both validation cohorts, discrimination is broadly stable across missingness strata, with no monotonic decline as missingness increases. In external validation, AUC ranges from 0.80–0.84 and AUPRC from 0.50–0.65; in temporal validation, AUC ranges from 0.78–0.86 and AUPRC from 0.45–0.71. Even in the 20–30% missingness bin, performance remains comparable to lower-missingness bins. These findings suggest the model is robust to routine EHR missingness, consistent with the tree-based handling of missing values. Estimates at higher missingness levels should be interpreted cautiously because those bins contain fewer windows, but there is no evidence of systematic performance decay within the observed range (≤30% missingness).

	External			Temporal		
	%windows	AUC	AUPRC	%windows	AUC	AUPRC
0-5	43.4	0.81	0.51	40.0	0.78	0.45
5-10	12.7	0.82	0.65	15.5	0.83	0.71
10-15	8.4	0.81	0.59	15.0	0.86	0.65
15-20	4.3	0.84	0.62	13.6	0.85	0.63
20-30*	31.3	0.80	0.50	16.0	0.82	0.55

Table S5. Discrimination by overall feature missingness in the prediction window. Windows are grouped by the percentage of missing values across model input features (0–5%, 5–10%, 10–15%, 15–20%, 20–30%). For each bin, the table reports the share of windows and model performance (AUC, AUPRC) in external and temporal validation cohorts. *No windows exceeded 30% missingness.

In Table S6, we assess the sensitivity analysis using alternative AKI definitions and focus on discrimination and precision-recall performance only. When AKI is defined by urine output (UO), AUC is 0.824 in temporal validation and 0.835 in external validation, while AUPRC is 0.538 and 0.471, respectively. Under creatinine-only (SCr) definitions, AUC is slightly lower in both cohorts (temporal 0.813; external 0.796), whereas AUPRC is mixed: it is modestly higher in temporal validation (0.569) but slightly lower in external validation (0.458) compared with UO-based AKI. Overall, these results show stable discrimination across AKI criteria, with only small shifts in AUPRC, consistent with differences in event prevalence and labelling strictness between UO- and creatinine-based definitions.

**Cohort**	**AKI definition**	**AUC**	**AUPRC**
Temporal	Urine output (UO)	0.824	0.538
External	Urine output (UO)	0.835	0.471
Temporal	Creatinine (SCr)	0.813	0.569
External	Creatinine (SCr)	0.796	0.458

Table S6. Discrimination by AKI definition in temporal and external validation cohorts. AUC and AUPRC are reported for urine output (UO)-based and creatinine (SCr)-based AKI labels.

Calibration was evaluated in detail. For each dataset (cross-validation, temporal, and geographical), we estimated the calibration intercept and calibration slope by fitting a logistic calibration model with the outcome as the dependent variable and the logit of the predicted probabilities as the independent variable; an intercept of 0 and a slope of 1 indicate perfect calibration. We also assessed calibration using calibration-in-the-large (CITL) and expected calibration error (ECE). Overall calibration was additionally quantified using the Brier score. To obtain well-calibrated probabilities, we applied Platt scaling to the raw XGBoost outputs, fitting a logistic recalibration model on out-of-fold predictions from the cross-validation procedure and then applying the resulting transformation to predictions in the temporal and geographical validation sets. We also constructed calibration plots (reliability diagrams) by grouping patients into quantiles of predicted risk and comparing mean predicted versus observed event rates within each group, providing a visual assessment of under- or over-prediction across the risk spectrum. (Figure S2)

To evaluate potential clinical usefulness, we performed decision curve analysis (DCA) using the calibrated probabilities. For a range of clinically relevant threshold probabilities, we calculated the net benefit of using the model to guide decisions and compared it against default “treat-all” and “treat-none” strategies (Figure S3). This allowed us to assess whether, and over which threshold ranges, the model would be expected to confer greater net clinical benefit than these default strategies, again in line with TRIPOD+AI recommendations for evaluating clinical utility. Finally, we explored predictor-level contributions using SHAP values, computed with a tree-based explainer applied to the final boosted tree ensemble. SHAP values decompose each individual prediction into additive contributions from each predictor on the log-odds scale, enabling both global summaries (e.g. variable importance ordered by the average absolute SHAP value) and local explanations for specific patients. In addition to SHAP-based interpretability, we also extracted the built-in XGBoost feature importance based on gain, which summarises, for each predictor, the average improvement in the optimisation objective attributable to splits on that variable (Figure S4). Together, SHAP analyses and gain-based feature importance were used to characterise which predictors most strongly influenced the model’s predictions and to verify that the learned relationships were clinically plausible.

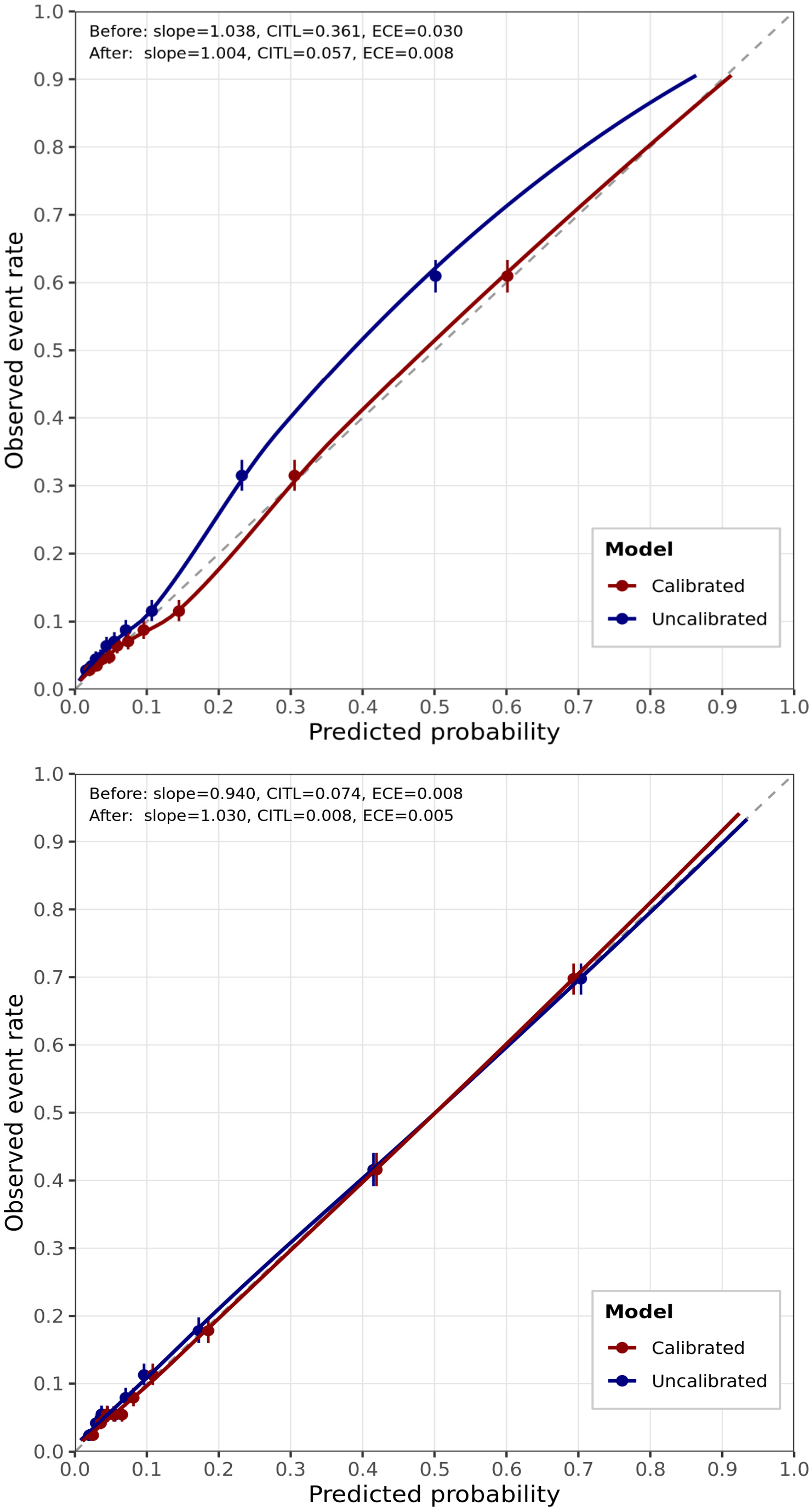

Figure S2. Calibration curves for the final model in external validation (upper panel) and temporal validation (lower panel). Observed event rates are plotted against predicted probabilities; the 45° line indicates perfect calibration. The uncalibrated model (blue) is compared with Platt-calibrated predictions (red). Reported statistics summarize calibration before and after recalibration: slope, calibration-in-the-large (CITL), and expected calibration error (ECE).

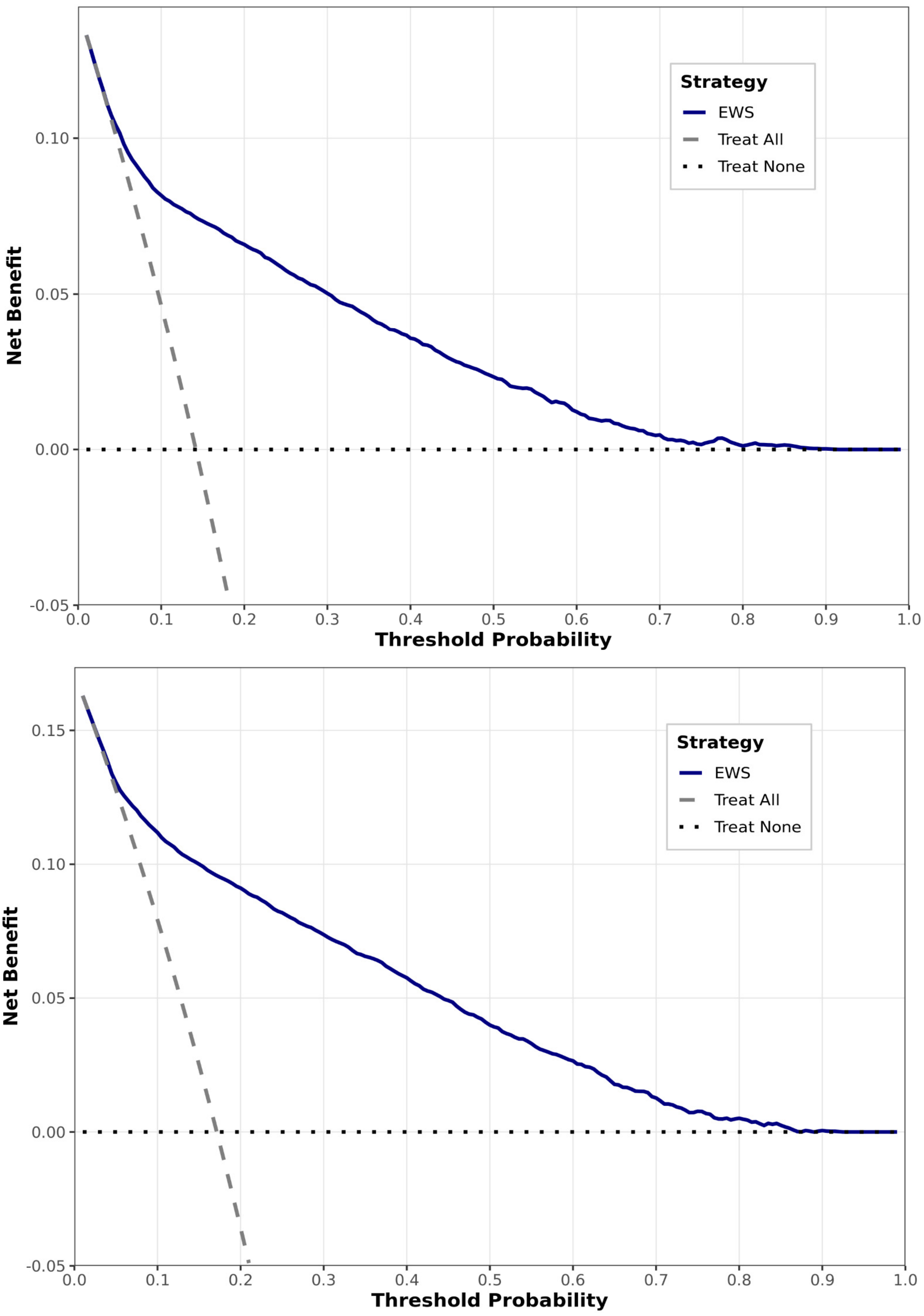

Figure S3. Decision curve analysis for external validation (upper panel) and temporal validation (lower panel). Net benefit is plotted across threshold probabilities for the early-warning score (EWS, solid blue), compared with "treat all" (dashed gray) and "treat none" (dotted black), for prediction of 24-h AKI. Curves above both reference strategies indicate clinical utility.

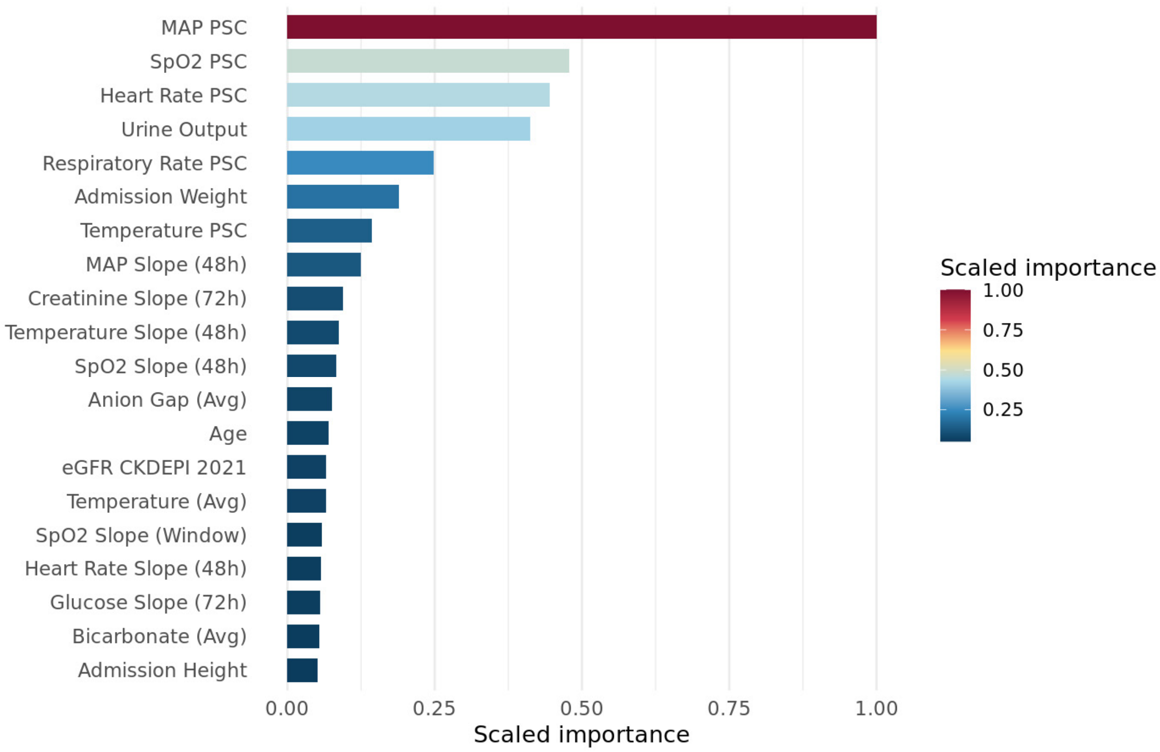

Figure S4. Gain-based feature importance from the final XGBoost model (top 20 predictors). Bar length shows each feature's contribution to loss reduction (gain), scaled so the most important feature equals 1.0. Colour encodes the same scaled importance. Abbreviations: PSC, piecewise slope change.

To further examine performance on external data, we quantified hospital-level discrimination for the monotonic XGBoost model within the eICU Northeast cohort and summarised results using a random-effects meta-analytic framework. For each hospital, we computed the AUC with corresponding 95% confidence interval and displayed these estimates in a forest plot (Figure S5). The random-effects model yielded a pooled AUC of 0.82 (95% CI 0.81–0.84), indicating consistently good discrimination across sites. Between-hospital heterogeneity was modest (τ^2 = 0.0001, I^2 = 39.97%, H^2 = 1.665), and Cochran's Q-test did not provide strong evidence against homogeneity (p = 0.1696). Together, these findings suggest that observed variation in hospital-level AUCs is limited and largely compatible with sampling variability, supporting the geographic robustness of the model's performance within the external eICU setting.

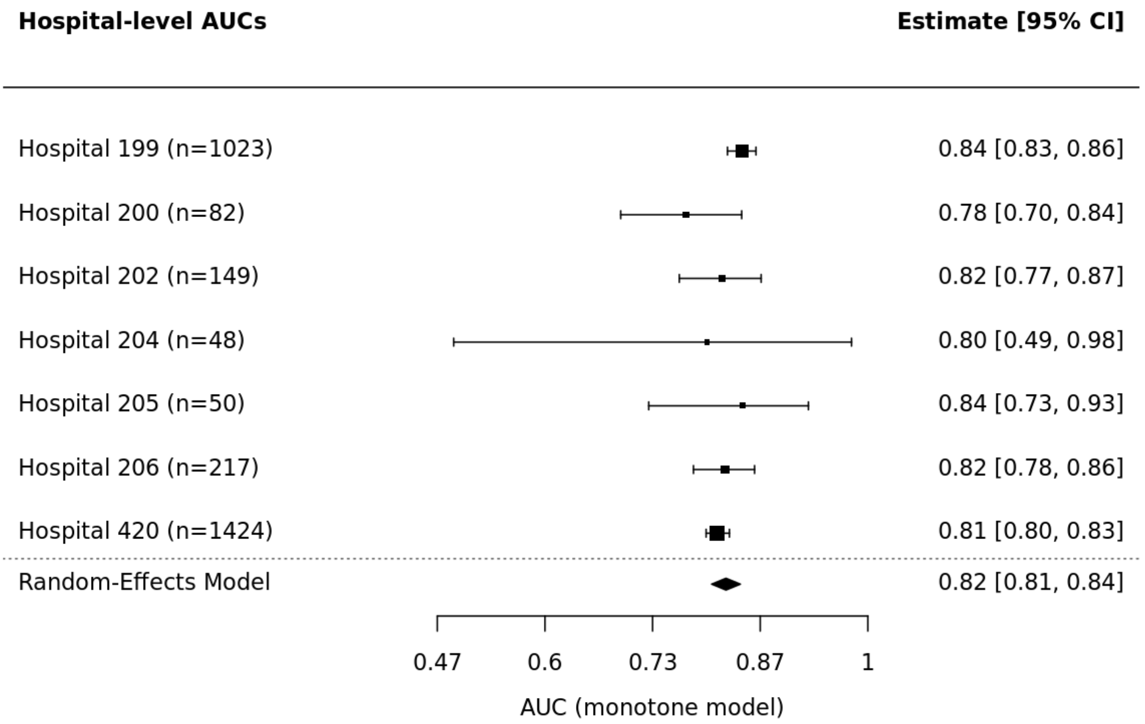

Figure S5. Hospital-level discrimination in the external eICU cohort.

To characterize how early the model warns before AKI onset, we performed event-level detection analyses using the first alert that precedes each AKI event. For each ICU stay with incident AKI, we identified the first AKI window (event time) and the earliest alert prior to that event, then defined lead time as the difference between the event time and the first alert time (hours). Detection rates were computed at prespecified thresholds (>6, >12, >18, >24 h), reporting the fraction of AKI events with lead time at or beyond each threshold. In addition, we summarized detected events across exclusive "guaranteed lead-time" bins (2–6, 6–12, 12–18, 18–24 h), which align with the 2-h gap between the feature window and outcome horizon and provide a distributional view of how much advance warning was achieved for events that were detected. Across validation cohorts (Figure S6), detection rates decreased gradually as the required lead time increased. In temporal validation, 30.6% of events were detected with >6 h lead time, falling to 24.4% at >12 h, 20.1% at >18 h, and 16.0% at >24 h. External validation showed similar or slightly higher detection at each threshold (32.8%, 27.3%, 23.2%, and 19.4%, respectively). These results indicate that roughly one-third of AKI events were flagged more than 6 hours in advance, and approximately one-sixth to one-fifth were detected at least 24 hours before onset.

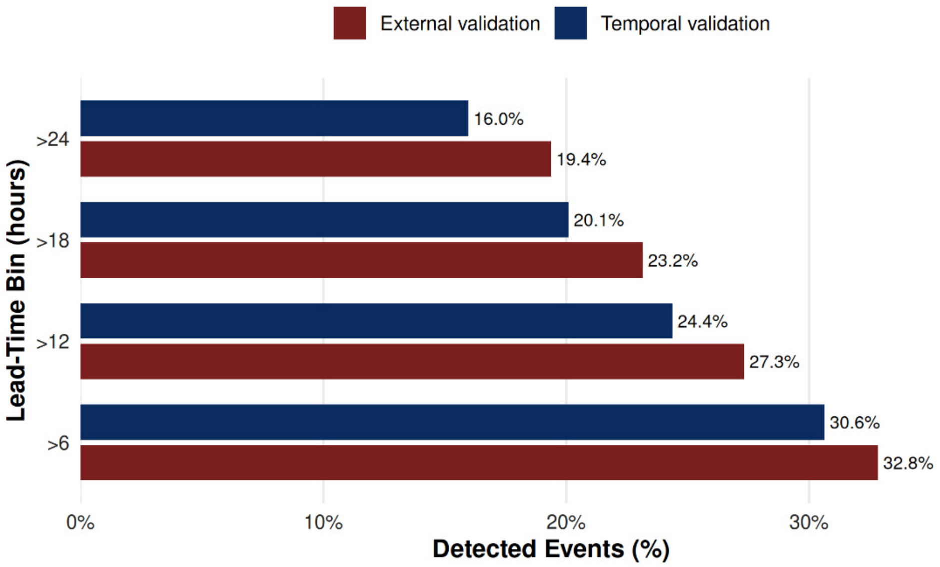

Figure S6. Event-level lead-time detection for temporal and external validation cohorts. The first alert preceding each AKI event is used to compute lead time; panels summarize detection rates at prespecified thresholds (>6, >12, >18, >24 h).

We also quantified alert burden and false-positive behavior at the prespecified alert threshold used in the main analyses. Alert burden metrics (Table S7) included total alerts, alerts per patient-day and per 12-h shift, time under alert (both raw and capped at length of stay), repeat-alert rate, and the proportion of stays with any or multiple alerts. Temporal validation yielded 4,252 alerts and external validation 3,538 alerts, corresponding to 0.19 and 0.21 alerts per patient-day (0.096 and 0.103 per 12-h shift). Repeat alerts accounted for 16.1% and 12.4% of alerts, and 20.95% and 15.77% of stays had at least two alerts in temporal and external cohorts, respectively.

Cohort	Alerts per patient-day	Alerts per 12-h shift	Mean time under alert (h)	Fraction time under alert (%)	Repeat alerts (%)	Stays with >=2 alerts (%)
Temporal (MIMIC-IV)	0.191	0.096	7.625	4.8	16.1	21.0
External (eICU)	0.206	0.103	7.093	5.1	12.4	15.8

Table S7. Alert burden metrics at the prespecified alert threshold (0.125) in temporal (MIMIC-IV) and external (eICU) validation cohorts. Rates are reported per patient-day and per 12-hour shift; time-under-alert is length-of-stay capped; percentages refer to alert-level or stay-level proportions as labeled.

For false positives, we classified alerts not followed by AKI within 24 h by the time to the next AKI event (24–48 h, 48–72 h, >72 h) or by the absence of any subsequent AKI; the distribution of these categories is reported in Table S8.

Reason	External	Temporal
Alert is 24-48h earlier	33.1%	37.4%
Alert is 48-72h earlier	18.9%	18.3%
Alert is >72 h earlier	15.8%	10.3%
No AKI experienced after alert	32.2%	34.1%

Table S8. Distribution of false-positive alerts by time to the next AKI event in temporal and external validation cohorts. Alerts not followed by AKI within the 24-h prediction horizon are grouped into 24–48 h, 48–72 h, >72 h, or no subsequent AKI. Values are percentages of false-positive alerts within each cohort.

Subgroup	Sensitivity	False negative rate	Specificity	False positive rate	CITL (Intercept)	Calibration slope	Brier score
**Sex**							
**Female**	75.5 (74.6–78.4)	24.5 (19.6–28.4)	83.4 (82.6–87.2)	16.6 (12.8–17.4)	−0.05 (−0.12 to +0.02)	0.96 (0.90–1.03)	0.09 (0.08–0.09)
**Male (ref)**	74.2 (72.2–78.6)	25.8 (18.4–29.8)	82.8 (81.5–86.6)	17.2 (13.4–18.5)	+0.01 (−0.05 to +0.06)	0.98 (0.93–1.04)	0.09 (0.09–0.10)
**Age group**							
**<50 (ref)**	76.2 (75.0–80.5)	23.8 (19.5–25.0)	83.8 (82.5–90.3)	16.2 (9.7–17.5)	−0.09 (−0.20 to +0.02)	0.94 (0.85–1.04)	0.074 (0.07–0.08)
**50–69**	75.4 (73.0–81.3)	24.6 (18.7–27.0)	83.2 (82.1–86.9)	16.8 (13.1–17.9)	+0.01 (−0.06 to +0.06)	0.98 (0.92–1.04)	0.091 (0.09–0.09)
**≥70**	74.8 (72.8–79.1)	25.2 (20.9–27.2)	82.9 (81.2–84.8)	17.1 (15.2–18.8)	+0.08 (+0.01 to +0.15)	0.92 (0.86–0.99)	0.108 (0.10–0.11)
**Race/ethnicity**							
**White (ref)**	76.0 (74.4–81.0)	24.0 (19.0–25.6)	83.1 (82.3–86.9)	16.9 (13.1–17.7)	+0.01 (−0.05 to +0.05)	0.98 (0.92–1.03)	0.093 (0.09–0.10)
**Black**	75.5 (73.2–82.2)	24.5 (17.8–26.8)	85.4 (83.5–87.2)	14.6 (12.8–16.5)	−0.04 (−0.14 to +0.06)	0.95 (0.86–1.06)	0.096 (0.09–0.10)
**Asian**	74.8 (72.0–84.2)	25.2 (15.8–28.0)	83.0 (82.3–89.5)	17.0 (10.5–17.7)	−0.07 (−0.22 to +0.09)	0.97 (0.82–1.12)	0.084 (0.08–0.09)
**Hispanic**	73.2 (68.0–83.0)	26.8 (17.0–32.0)	84.5 (81.0–90.0)	15.5 (10.0–19.0)	−0.15 (−0.33 to +0.05)	0.90 (0.74–1.08)	0.086 (0.08–0.10)

Table S9. Threshold-based error rates by subgroup at the prespecified alert threshold. Threshold-based performance is reported for each prespecified subgroup at the operating threshold used in the main analysis (predicted AKI risk ≥ 0.125). Metrics include sensitivity (true positive rate, TPR), specificity (true negative rate, TNR), false negative rate (FNR = 1 − sensitivity), and false positive rate (FPR = 1 − specificity). AKI prevalence is the observed event rate within each subgroup. Values are shown as point estimates with 95% confidence intervals obtained via patient-level bootstrap resampling (resampling at the patient/admission level to preserve within-patient correlation across time windows). FNR is emphasized because missed AKI risk may lead to disproportionate clinical harm relative to false positive alerts.

Proposed Clinical Protocol for AKI Early Warning Response

To maximize clinical impact, we recommend a standardized response when the AKI Early Warning Score triggers a high-risk alert. The following protocol synthesizes best practices (including KDIGO guideline recommendations) into a practical checklist for the care team.

EWS Risk Thresholds: The AKI EWS outputs a probability (%) that the patient will develop AKI within the next 24 hours. We define two actionable risk tiers:

- High Risk – Predicted risk > =12.5%.
- Low-Medium Risk – Predicted risk < 12.5%.

Since AKI is highly prevalent in ICU patients and our interventions are triggered once risk exceeds a single actionable threshold, we use two tiers rather than three (an “intermediate” tier would not change management), but deliberately label the non-high group as “low–medium” rather than simply “low” to reflect that some patients in this band still carry a meaningful AKI risk.

When feasible, we recommend calibrating the probabilities and adjusting thresholds based on retrospective data to prevent sudden shifts.

When an alert is issued, proceed as follows:

- Acknowledge Alert and Determine Risk Level:

- Identify whether the alert is Low-Medium Risk or High risk
- High Risk: This warrants urgent action – immediate evaluation and then intervention if needed (see below).
- Low-Medium Risk: If no clinical-based suspicion of AKI; surveillance only. Ensure accurate UO capture.

Communicate to the team: e.g., “AKI alert for Bed 7 – high risk” so that bedside nurse, responsible MD, and team are aware and can mobilize.

1. **Alert Notification and Triage:**

i. Team Communication: The moment the EWS risk exceeds the predetermined threshold (e.g. 12.5% probability of AKI within 24h), an alert is issued in the ICU electronic record and/or via pager. The bedside nurse and responsible ICU physician are notified in real time.

ii. Initial Triage: Assign a team member (ICU fellow or bedside nurse) to promptly reassess the patient’s current status. Verify whether AKI is already present (check latest creatinine/urine output) and ensure there are no emergent issues requiring immediate intervention (e.g. severe hypotension or sepsis needing urgent attention).

2. **Confirm and Monitor Renal Function:**

i. Laboratory Re-check: Order a repeat serum creatinine and review the trend. If no recent lab within the past 6 hours, obtain a current BMP (basic metabolic panel) to confirm creatinine and electrolytes status.

ii. Urine Output Monitoring: Ensure accurate urine output measurement. If the patient does not have a Foley catheter, consider placing one for precise output monitoring, especially if oliguria is suspected. Initiate strict intake-output charting.

iii. Point-of-Care Testing: If available, perform a urinalysis (dipstick or microscopy) to check for hematuria, proteinuria or casts, which might indicate intrinsic kidney injury. Consider point-of-care renal ultrasound to assess for bladder retention or hydronephrosis (obstruction).

iv. Electrolyte Danger Check (Immediate): If potassium is elevated or rising, initiate standard ICU hyperkalemia precautions and treatment as indicated (repeat ECG, temporizing measures such as calcium for membrane stabilization when indicated, insulin/dextrose, beta-agonist, potassium binders where appropriate, and early escalation if refractory), and reassess frequently given that worsening hyperkalemia is a key trigger for urgent RRT.

3. **Optimize Hemodynamics and Perfusion:**

i. Blood Pressure Support: Check hemodynamics, with particular attention to mean arterial pressure (MAP). If MAP is below the target (commonly 65 mmHg or an individualized goal), intervene to improve renal perfusion. This may include cautious fluid boluses if the patient is volume responsive (assess using dynamic indices or bedside ultrasound) and/or adjustment of vasopressors.Aim to avoid prolonged hypotension, as even brief MAP drops can precipitate AKI. In patients with chronic hypertension or suspected renal hypoperfusion, consider targeting a higher MAP than 65 mmHg (e.g., 80–85 mmHg) when clinically appropriate, rather than applying a single MAP target to all patients. When vasopressors are required, specify a default hierarchy (e.g., norepinephrine first-line in distributive shock, with consideration of adding vasopressin as a second-line agent in selected patients) and document the rationale for kidney-perfusion goals.

ii. Cardiac Output: Evaluate signs of low cardiac output (cool extremities, lactate levels, echocardiogram if available). If cardiac output is suspected to be inadequate, consider inotropic support or advanced monitoring. Optimizing cardiac index can further enhance renal blood flow.

iii. Avoid Venous Congestion: Ensure the patient is not fluid-overloaded to the point of raising central venous pressure excessively, as venous congestion can impair renal perfusion.

4. Review and Adjust Medications:

i. Nephrotoxin Audit: Immediately review all medications and recent exposures. Discontinue or pause nephrotoxic drugs where possible. This includes NSAIDs, high-dose diuretics (if the patient is not fluid-overloaded), ACE inhibitors/ARBs, aminoglycosides, vancomycin (if safe alternatives or dose adjustments can be made), iodinated contrast media, etc. If a contrast study is necessary, ensure appropriate preventive measures (hydration, minimum contrast dose) are in place.

ii. Therapeutic Drug Monitoring: For essential medications that carry nephrotoxicity risk (e.g. vancomycin, amphotericin B), perform therapeutic drug level monitoring to adjust dosing and minimize toxicity. Consider pharmacy consult for optimized dosing of antibiotics and adjust any renally cleared drugs to current GFR estimates. Because creatinine-based eGFR/CrCl can be unreliable in critical illness (non–steady state, altered volume of distribution), do not rely on a single estimated GFR value; instead, base dosing on trends, urine output, measured drug levels when available, and reassess dosing after each meaningful creatinine/UO change. If CRRT or intermittent RRT is initiated, explicitly trigger a “medication re-dosing review” (antibiotics, antiepileptics, sedatives, anticoagulants, etc.), because clearance differs substantially by modality and settings.

iii. Renal Protection Agents: Although no pharmacologic prophylaxis has definitively proven to prevent AKI, ensure the patient is on appropriate renal dose dopamine – not recommended (avoid, as per guidelines). Instead, consider N-acetylcysteine if contrast exposure is imminent (acknowledging mixed evidence), and ensure adequate systemic anticoagulation if at risk for microvascular thrombosis (e.g. DIC or CRRT filter clotting, according to ICU protocol).

5. Fluid Management:

i. Assess Volume Status: Conduct a focused fluid status assessment. Examine clinical signs (mucous membranes, edema), check fluid balance over past 24h, and consider ultrasound assessment (IVC variability, lung ultrasound B-lines, VExUS score if expertise available).

ii. Fluid Resuscitation or Removal: If the patient appears volume-depleted (e.g. low JVP, flat IVC, hypotension with tachycardia), administer a balanced crystalloid bolus and reassess output and hemodynamics frequently. When choosing fluids, prefer balanced crystalloids over normal saline for substantial resuscitation when feasible, given concerns that high chloride load may contribute to hyperchloremic metabolic acidosis and potentially adverse renal haemodynamics.

iii. Avoid starch or colloid solutions in AKI-risk patients due to possible harm. Conversely, if volume overload is present (positive fluid balance with high CVP and edema), cautious diuresis may be warranted to relieve venous pressure – provided perfusion is maintained. The goal is a euvolemic state optimizing kidney perfusion without congestion.

iv. Ongoing Monitoring: Continue to track hourly urine output after interventions. If oliguria persists (<0.5 mL/kg/h for 6 hours) despite optimization, escalate care (e.g. consider invasive hemodynamic monitoring or nephrology consult as below).

6. Investigations for Cause of AKI:

i. Evaluate for Acute Insults: Systematically consider common AKI precipitants. Check for any signs of sepsis or infection (fever, cultures) and treat promptly if present. Inspect recent labs for hemolysis or rhabdomyolysis (LDH, haptoglobin, CK levels) which may need specific interventions. Ensure abdominal compartment syndrome is not present in surgical patients (bladder pressure measurement if concern).

ii. Imaging: If obstruction is in the differential (e.g. new anuria, history of stones, or known single kidney), obtain a renal ultrasound to rule out obstructive uropathy, as relieving an obstruction (e.g. via catheter or stent) can swiftly reverse AKI.

iii. Biomarkers: (Optional) If available, consider measuring stress biomarkers such as [TIMP-2]*[IGFBP7] which can indicate early kidney stress, to corroborate the high-risk status.

7. Specialist Consultation:

i. Nephrology Consult: In high-risk situations or if moderate AKI (KDIGO stage 2 or higher) is already present or imminent, involve a nephrologist early. Consultation is advised if despite initial interventions creatinine continues to rise or urine output remains very low. A specialist can help tailor further measures (e.g. electrolyte management, considering renal replacement therapy (RRT) initiation if criteria arise, or dosing adjustments for renally excreted drugs). Early nephrology involvement has been associated with better preparation for potential dialysis and can facilitate timely RRT if needed. In the consult trigger, explicitly frame RRT initiation around urgent indications (e.g., refractory hyperkalemia, severe/refractory metabolic acidosis, diuretic-resistant fluid overload with hypoxemia, or uremic complications) rather than initiating RRT solely because predicted risk is high. Also document that “accelerated/early” RRT in the absence of urgent indications has not consistently improved mortality in major trials, while excessive delay once complications evolve may be harmful—supporting a balanced, indication-driven approach.

ii. Critical Care Team Huddle: If the AKI risk alert persists or worsens over subsequent model updates, call for a multidisciplinary team huddle (intensivist, nephrologist, pharmacist, senior nurse) to re-evaluate the case and ensure all preventative actions are being addressed.

8. Education and Documentation:

i. Communicate with Patient/Family: If appropriate, inform the patient (or family) that the patient is at risk for kidney injury. Emphasize the proactive steps being taken. This helps manage expectations and engages them in, for example, avoiding NSAIDs or adjusting fluid intake if applicable.

ii. Document AKI Risk and Response: In the medical record, document the EWS alert and the actions taken (e.g. “AKI risk alert at 40% – initiated KDIGO bundle: held nephrotoxics, optimized MAP with fluids/vasopressor, will monitor U/O closely, nephrology consulted”). This ensures clear communication across shifts and creates a record for quality improvement review.

9. Follow-Up and Reevaluation:

i. Frequent Reassessment: Continuously monitor kidney function trends over the next 24–48 hours. Repeat labs (creatinine, BUN, electrolytes) at least daily (or more frequently if trending upward or if interventions like diuresis are initiated). Monitor urine output every hour. The EWS will update every 6 hours; note whether the risk score decreases in response to interventions (an improving score may reinforce that measures are working).

ii. Adjust Plan as Needed: If risk remains high or kidney function is deteriorating, escalate support. This may include starting renal replacement therapy in ICU for worsening metabolic status or volume overload unresponsive to diuretics, per standard AKI management protocols. Conversely, if the risk score drops significantly and the patient remains stable, continue routine care but maintain vigilance until the risk period passes. If metabolic acidosis is present and not yet an absolute indication for RRT, consider an explicit step for alkali therapy (e.g., bicarbonate) where appropriate per ICU protocol and etiology, while monitoring for sodium/volume consequences and reassessing the need for RRT if acidosis remains refractory. If hyperphosphatemia develops, define a management approach that accounts for nutrition status and RRT modality (dietary adjustment when feasible, RRT optimization if on CRRT, and cautious/individualized consideration of phosphate binders recognizing limited evidence and practical constraints in the ICU).

iii. Chart Review for Outcome: If the patient does develop AKI despite preventive measures, conduct a post-AKI review. Determine if any modifiable factors could have been addressed earlier or if the alert could have been acted on more aggressively – this feedback loop will help refine the protocol and the threshold settings of the EWS to further improve performance over time.

Section/Topic	Item	Development / evaluation[1]	Checklist item	Reported on page
**TITLE**				
Title	1	D;E	Identify the study as developing or evaluating the performance of a multivariable prediction model, the target population, and the outcome to be predicted	1
**ABSTRACT**				
Abstract	2	D;E	See TRIPOD+AI for Abstracts checklist	1
**INTRODUCTION**				
Background	3a	D;E	Explain the healthcare context (including whether diagnostic or prognostic) and rationale for developing or evaluating the prediction model, including references to existing models	2
	3b	D;E	Describe the target population and the intended purpose of the prediction model in the context of the care pathway, including its intended users (e.g., healthcare professionals, patients, public)	2
	3c	D;E	Describe any known health inequalities between sociodemographic groups	2
Objectives	4	D;E	Specify the study objectives, including whether the study describes the development or validation of a prediction model (or both)	3
**METHODS**				
Data	5a	D;E	Describe the sources of data separately for the development and evaluation datasets (e.g., randomised trial, cohort, routine care or registry data), the rationale for using these data, and representativeness of the data	3/ 1^s
	5b	D;E	Specify the dates of the collected participant data, including start and end of participant accrual; and, if applicable, end of follow-up	3/ 1^s
Participants	6a	D;E	Specify key elements of the study setting (e.g., primary care, secondary care, general population) including the number and location of centres	3/ 1^s
	6b	D;E	Describe the eligibility criteria for study participants	3/ 1^s, 2^s
	6c	D;E	Give details of any treatments received, and how they were handled during model development or evaluation, if relevant	-
Data preparation	7	D;E	Describe any data pre-processing and quality checking, including whether this was similar across relevant sociodemographic groups	4/ 1^s-3^s
Outcome	8a	D;E	Clearly define the outcome that is being predicted and the time horizon, including how and when assessed, the rationale for choosing this outcome, and whether the method of outcome assessment is consistent across sociodemographic groups	3/ 3^s
	8b	D;E	If outcome assessment requires subjective interpretation, describe the qualifications and demographic characteristics of the outcome assessors	3/ 3^s
	8c	D;E	Report any actions to blind assessment of the outcome to be predicted	-
Predictors	9a	D	Describe the choice of initial predictors (e.g., literature, previous models, all available predictors) and any pre-selection of predictors before model building	4/ 3^s-5^s
	9b	D;E	Clearly define all predictors, including how and when they were measured (and any actions to blind assessment of predictors for the outcome and other predictors)	3^s-5^s
	9c	D;E	If predictor measurement requires subjective interpretation, describe the qualifications and demographic characteristics of the predictor assessors	3^s-5^s
Sample size	10	D;E	Explain how the study size was arrived at (separately for development and evaluation), and justify that the study size was sufficient to answer the research question. Include details of any sample size calculation	S
Missing data	11	D;E	Describe how missing data were handled. Provide reasons for omitting any data	4/ 1^s, 3^s
Analytical methods	12a	D	Describe how the data were used (e.g., for development and evaluation of model performance) in the analysis, including whether the data were partitioned, considering any sample size requirements	5/ 1^s, 3^s
	12b	D	Depending on the type of model, describe how predictors were handled in the analyses (functional form, rescaling, transformation, or any standardisation).	5/ 3^s
	12c	D	Specify the type of model, rationale[2], all model-building steps, including any hyperparameter tuning, and method for internal validation	5/ 6^s-8^s
	12d	D;E	Describe if and how any heterogeneity in estimates of model parameter values and model performance was handled and quantified across clusters (e.g., hospitals, countries). See TRIPOD-Cluster for additional considerations[3]	6^s-7^s
	12e	D;E	Specify all measures and plots used (and their rationale) to evaluate model performance (e.g., discrimination, calibration, clinical utility) and, if relevant, to compare multiple models	5/ 6^s-8^s
	12f	E	Describe any model updating (e.g., recalibration) arising from the model evaluation, either overall or for particular sociodemographic groups or settings	5/ 8^s
	12g	E	For model evaluation, describe how the model predictions were calculated (e.g., formula, code, object, application programming interface)	5/ 6^s
Class imbalance	13	D;E	If class imbalance methods were used, state why and how this was done, and any subsequent methods to recalibrate the model or the model predictions	3^s, 6^s
Fairness	14	D;E	Describe any approaches that were used to address model fairness and their rationale	5/ 3^s-7^s
Model output	15	D	Specify the output of the prediction model (e.g., probabilities, classification). Provide details and rationale for any classification and how the thresholds were identified	5-6, 11
Training versus evaluation	16	D;E	Identify any differences between the development and evaluation data in healthcare setting, eligibility criteria, outcome, and predictors	6-10
Ethical approval	17	D;E	Name the institutional research board or ethics committee that approved the study and describe the participant-informed consent or the ethics committee waiver of informed consent	5
**OPEN SCIENCE**				

Funding	18a	D;E	Give the source of funding and the role of the funders for the present study	20
Conflicts of interest	18b	D;E	Declare any conflicts of interest and financial disclosures for all authors	21
Protocol	18c	D;E	Indicate where the study protocol can be accessed or state that a protocol was not prepared	17/ 12[s]-15[s]
Registration	18d	D;E	Provide registration information for the study, including register name and registration number, or state that the study was not registered	1[s]
Data sharing	18e	D;E	Provide details of the availability of the study data	21
Code sharing	18f	D;E	Provide details of the availability of the analytical code[4]	21
**PATIENT & PUBLIC INVOLVEMENT**				
Patient & Public Involvement	19	D;E	Provide details of any patient and public involvement during the design, conduct, reporting, interpretation, or dissemination of the study or state no involvement.	1[s]
**RESULTS**				
Participants	20a	D;E	Describe the flow of participants through the study, including the number of participants with and without the outcome and, if applicable, a summary of the follow-up time. A diagram may be helpful.	5/ 2[s]
	20b	D;E	Report the characteristics overall and, where applicable, for each data source or setting, including the key dates, key predictors (including demographics), treatments received, sample size, number of outcome events, follow-up time, and amount of missing data. A table may be helpful. Report any differences across key demographic groups.	6-10
	20c	E	For model evaluation, show a comparison with the development data of the distribution of important predictors (demographics, predictors, and outcome).	6-10
Model development	21	D;E	Specify the number of participants and outcome events in each analysis (e.g., for model development, hyperparameter tuning, model evaluation)	5-10/ 2[s]
Model specification	22	D	Provide details of the full prediction model (e.g., formula, code, object, application programming interface) to allow predictions in new individuals and to enable third-party evaluation and implementation, including any restrictions to access or re-use (e.g., freely available, proprietary)[5]	21
Model performance	23a	D;E	Report model performance estimates with confidence intervals, including for any key subgroups (e.g., sociodemographic). Consider plots to aid presentation.	10-14
	23b	D;E	If examined, report results of any heterogeneity in model performance across clusters. See TRIPOD Cluster for additional details[3].	10-11
Model updating	24	E	Report the results from any model updating, including the updated model and subsequent performance	-
**DISCUSSION**				
Interpretation	25	D;E	Give an overall interpretation of the main results, including issues of fairness in the context of the objectives and previous studies	15-17
Limitations	26	D;E	Discuss any limitations of the study (such as a non-representative sample, sample size, overfitting, missing data) and their effects on any biases, statistical uncertainty, and generalizability	15-17, 20
Usability of the model in the context of current care	27a	D	Describe how poor quality or unavailable input data (e.g., predictor values) should be assessed and handled when implementing the prediction model	15,18
	27b	D	Specify whether users will be required to interact in the handling of the input data or use of the model, and what level of expertise is required of users	15, 17-19
	27c	D;E	Discuss any next steps for future research, with a specific view to applicability and generalizability of the model	20
